# Advances in Electronic-Nose Technologies Developed for Biomedical Applications

**DOI:** 10.3390/s110101105

**Published:** 2011-01-19

**Authors:** Alphus D. Wilson, Manuela Baietto

**Affiliations:** 1 Southern Hardwoods Laboratory, Center for Bottomland Hardwoods Research, Southern Research Station, USDA Forest Service, 432 Stoneville Road, Stoneville, MS 38776, USA; 2 Dipartimento di Produzione Vegetale, Università degli Studi di Milano, Via Celoria 2, 20133 Milan, Italy; E-Mail: manuela.baietto@unimi.it

**Keywords:** artificial olfaction, disease diagnoses, electronic aroma detection, e-nose, healthcare applications

## Abstract

The research and development of new electronic-nose applications in the biomedical field has accelerated at a phenomenal rate over the past 25 years. Many innovative e-nose technologies have provided solutions and applications to a wide variety of complex biomedical and healthcare problems. The purposes of this review are to present a comprehensive analysis of past and recent biomedical research findings and developments of electronic-nose sensor technologies, and to identify current and future potential e-nose applications that will continue to advance the effectiveness and efficiency of biomedical treatments and healthcare services for many years. An abundance of electronic-nose applications has been developed for a variety of healthcare sectors including diagnostics, immunology, pathology, patient recovery, pharmacology, physical therapy, physiology, preventative medicine, remote healthcare, and wound and graft healing. Specific biomedical e-nose applications range from uses in biochemical testing, blood-compatibility evaluations, disease diagnoses, and drug delivery to monitoring of metabolic levels, organ dysfunctions, and patient conditions through telemedicine. This paper summarizes the major electronic-nose technologies developed for healthcare and biomedical applications since the late 1980s when electronic aroma detection technologies were first recognized to be potentially useful in providing effective solutions to problems in the healthcare industry.

## 1. Introduction

Rapid progress in the advancement of several key science areas including artificial intelligence, digital electronic sensors design, material sciences, microcircuitry design, software innovations, and electronic systems integration has stimulated the development of electronic sensor technologies applicable to many diverse areas of human activity. The conceptualization and production of electronic nose devices (initially called artificial noses) [1], improved and evolved since the early 1980s to mimic mammalian olfactory systems [2–4], have resulted in the creation of a remarkable new sector of sensor technology (generally referred to as artificial olfaction [5]) that has exploded with the production of many new potential applications largely resulting from the invention of numerous new types of olfactory-competent electronic sensors and sensor arrays. These sensor devices, termed electronic noses by Gardner and Bartlett [6] in 1994, are capable of detecting, identifying, and discriminating many types and sources of a wide diversity of chemical species and mixtures of compounds present in headspace volatiles of sampled air (derived from any source), including volatile organic compounds (VOCs) most commonly produced and released from such organic sources as living microbes and multicellular organisms. Recent advances in electronic-nose (e-nose) technologies, based on many different electronic aroma detection (EAD) principles and mechanisms [7], have made possible the development of a wide variety of new e-nose applications that have proven useful in a range of diverse commercial industries, including the agricultural, cosmetics, environmental, food, manufacturing, military, pharmaceutical, and regulatory sectors, and in many fields of applied sciences [8]. Advances in the use of electronic-nose instruments in biomedical applications are no exceptions. In fact, the development of e-nose applications in the healthcare industry arguably has grown more rapidly than any other industry due to higher available capitalization for products development in the healthcare industry, and greater economic incentives for the development and use of cheaper, high-performing electronic-sensor instruments. Greater demands on improvements in performance, effectiveness and lower costs of biomedical tools and instruments used in daily healthcare applications, also have resulted from increasing limitations on financial and physical resources as a consequence of increasing healthcare costs, budgetary cuts or constraints, changes in socio-economic factors and healthcare legislation, and increasing globalization of world economies that has allowed the sharing of technologies as a result of improved communications. Increasing demands on functional requirements of biomedical tools and machines has necessitated the development and application of new more efficient and intelligent diagnostic and aroma-mensurative devices that provide rapid and accurate qualitative and/or quantitative results with large-sample throughput and consistently-accurate results despite repeated use.

The characterization of abnormal chemical compounds associated with human diseases, particularly microbial volatiles of pathogenic origin, has been keenly investigated since the development of gas chromatography (GC) and mass spectroscopy (MS) instruments. Davies and Hayward [9] identified acetylcholine and proposed that it may be a precursor of trimethylamine, a possible early biochemical marker of urinary tract infections caused by *Proteus* and *Klebsiella* species. Grametbauer *et al.* [10] investigated the metabolic profiles of 189 strains of 33 bacterial species and found a wide diversity of volatile compounds was produced. They concluded that it was not possible to completely differentiate between strains of a species even when the volatile profiles showed distinct peaks. GC-MS analysis of VOCs from pathogenic bacteria such as *Pseudomonas aeruginosa*, *Proteus mirabilis*, *Klebsiella pneumoniae*, *Staphylococcus aureus*, and *Clostridium septicum* revealed that all of them produced complex odor patterns [11]. Similar results were reported from extensive studies of lactic acid-bacteria using GC techniques to distinguish between *Leuconostoc*, *Pediococcus*, and *Lactobacillus* species [12]. Mayakova *et al.* [13] developed a GC-MS method to analyze volatile fatty acids to detect post-operative infections caused by non-clostridial anaerobes in abdominal and gynecological surgery. Even though GC-MS analyses have enabled comprehensive studies and identifications of possible disease markers, these tools have not emerged as routine instruments for clinical diagnosis due to associated high operating costs, laborious and time-consuming sample-preparation methods, and requirements for significant training and expertise for effective operation and data interpretation [14,15]. In addition, the high complexity of volatile profiles detected using GC-MS methods precludes the ability to easily associate these chemical profiles to specific microbial or biotic sources because chemical profiles (unlike aroma profiles) are not considered as a whole in data analyses, for these instruments are designed to identify individual chemical compounds, not identify the complex sample mixture as a whole unit. GC-MS instruments have been very useful in facilitating our understanding of the biochemistry of human diseases and disorders, and identifying aberrant VOCs that may serve as diagnostic biomarkers of specific diseases and physiological or genetic disorders, but uses of these instruments should be limited to applications for which they were designed.

The limited applicability of GC-MS and similar analytical instruments in clinical diagnoses has prompted the need to develop simpler, cheaper, and more user-friendly diagnostic instruments for routine clinical applications. The invention of electronic noses made it possible, for the first time, to produce application-specific instruments that fulfill the needs of clinical requirements for routine use. For example, disease-diagnosis applications of e-noses provide the capabilities to more cheaply identify microbes from their precise mixtures of VOC headspace metabolites (including individual disease biomarkers) without having to identify all chemical species present in these usually complex mixtures. Each complex mixture of VOCs in a sampled headspace, derived from cultures or samples of a particular microbe, can be effectively associated with that specific microbial species through the recognition of a unique electronic aroma signature pattern (EASP), or aroma profile, that is exclusively associated only with that microbe (or even a specific strain), and predetermined in library recognition files that are produced and previously recorded from sensor outputs of the e-nose sensor array using known reference samples of various microbial species to produce a library of aroma profiles stored in corresponding databases [7].

Electronic-nose devices offer many potential uses and advantages for numerous biomedical applications as a result of a wide range of different operating principles associated with various e-nose instrument types and designs. The major strengths of e-nose devices are their versatile capabilities afforded by the potential abilities of developers to produce low-cost tools for particular applications with customized instrument designs with specific use-requirements, the high variety of individual sensors available for selection (from large sensor libraries) in designing instrument sensor arrays for specific applications, and the wide diversity of useful medical information that can be derived from aroma measurements and analyses of air samples obtained directly from human tissues, biopsies, or cultures. The following review thoroughly explores many of the most important and useful applications that electronic-nose applications offer to the healthcare and biomedical fields, including some exciting new potential applications derived from recent research findings of scientists throughout the world.

## 2. History of Aroma and Electronic Noses in Biomedicine

The use of unusual human odors or aromas as indicators of disease probably originated with Hippocrates, the father of medicine, around 400 BC [16]. A common diagnostic practice during that time was to pour human sputum on hot coals to generate a smell that provided some indication of human ailments. Traditional Chinese medicine also employed the power of olfactory diagnosis [17]. Even though these practices were used long before the development of the germ theory of disease, early medical practitioners clearly recognized that the presence of human diseases could change the odor of bodily excretions, including sweat, urine, vaginal fluid, and sputum. Liebig and Woehler [18] described the production of benzaldehyde by microorganisms in the early 19th century. The final connection between the foul odors of infected wounds and very similar odors produced in cultures of pathogenic bacteria, causal agents of many microbial diseases, was realized some time after conclusive proofs of the germ theory were demonstrated by Robert Koch in 1876 [19], based on experiments conducted on anthrax, the human and cattle disease caused by the bacterium *Bacillus anthracis*. Omelianski [20] reported naturally-liberated microbial odors associated with the accumulation of staling metabolic products such as organic acids and alcohols in cultures of *Mycobacterium tuberculosis* and *Pseudomonas aeruginosa*.

### 2.1. Early Uses of Descriptive Aromas in Healthcare Applications

Diagnosing human diseases based on the sense of smell, in addition to physical examinations, remained one of the most reliable methods used in bedside medicine for many years. The odors emitted by patients was once considered among the first major clues leading to an early diagnosis. The practice of evaluating effluvia (odor of patients), along with other extracorporeal information, was regarded with such importance that Fitzgerald and Tierney [21] (in 1982) summarized a range of different types of extracorporeal observations (clues) and supplemental diagnostic techniques that provided useful physical evidence for clinical diagnostic evaluations. They were most concerned that the art of olfactory and extracorporeal diagnosis would be lost to the next generation of physicians largely due to the lack of attention to and emphasis on application of these skills in contemporary office practice. To emphasize the value of preserving olfactory methods in the medical profession, they provided a number of different examples of aroma bioindicators for various types of disease. Other examples of descriptive aromas have been documented in numerous reports that associate connections between unusual specific human odors (or aromas) and particular human diseases and disorders. A list of some of these descriptive aromas that have been reported as positive indicators of specific diseases, physiological or metabolic disorders, and genetic anomalies are presented in Table 1. The long history of human odors being used for disease diagnosis by medical doctors attests to the usefulness and effectiveness of olfactory information in providing valuable clues for assessing patient conditions, and to the ingenuity and skill of medical practitioners in achieving accurate diagnoses without the aid of modern analytical equipment and chemical-detection devices now available for this purpose.

Some genetic disorders, caused by defects in amino acid metabolism, have been associated with particular, unusual smells. For example, certain hereditary metabolic diseases in children may be associated with specific odors in their excrement [41]. Maple syrup urine disease (MSUD) is a metabolic disorder caused by a gene defect that prevents the body from breaking down certain amino acids such as leucine, isoleucine, and valine through normal oxidative decarboxylation. This condition leads to a buildup of these amino acids in the blood. Persons with MSUD may produce urine that smells like maple syrup [23,26]. Similar diseases caused by enzyme deficiencies have a distinctive odor due to the accumulation of undecomposed metabolites in the body. Pediatricians have long associated phenylketonuria (PKU) in children with a mousy or barny smell in the urine [28]. Phenylketonuria is an inherited metabolic disorder caused by a deficiency of the specific enzyme phenylalanine hydroxylase (PAH). In the homozygous patient with PKU, their inability to synthesize PAH (in an active form) leads to hyperphenylalanineaemia and phenylpyruvic aciduria due to the failure to break down phenylalanine. Without the enzyme, levels of phenylalanine and closely-related compounds build up in the body to toxic levels. These substances are harmful to the central nervous system and can cause brain damage. Phenylacetic acid in sweat and urine produces a musty odor resembling sweaty locker-room towels [26]. Hypermethioninemia, due to deficiency of the enzyme S-adenosylhomocysteine hydrolase that causes abnormal handling of methionine, can produce a characteristic odor in the urine, sweat and breath that in some cases is sweet and in other cases likened to boiled cabbage [26]. Tanaka *et al.* [32] described the disease isovaleric acidemia of infants who emitted a smell similar to sweaty feet, owing to the accumulation of isovaleric acid caused by an enzymatic block in leucine metabolism. Other genetic disorders of amino acid metabolism, including histidinemia, cystinuria and tyrosinemia, cause the production of volatile oxidized amino-acid derivatives that may be detected by unusual odors in the human breath [42].

Several physiological orders also may be diagnosed by unusual body odors. Acromegaly, a chronic metabolic disorder caused by the production of excessive human growth hormone (HGH) by the pituitary gland leading to gradual enlargement of body tissues, is sometimes associated with a strongly offensive body odor and often accompanied by increased sweating and oily skin [22]. Trimethylaminuria (TMAU) is a hereditary metabolic disorder in which an individual is not able to convert trimethylamine into a compound called trimethylamine N-oxide. Trimethylaminuria is also known as fish odor syndrome or fish malodor syndrome due to the fishy odors that result from the accumulation of trimethylamine in the urine or volatilization after release from the skin [23]. Azotemia is a condition where the patient’s blood contains uncommon levels of urea, creatinine, and other compounds rich in nitrogen [24]. Prerenal azotemia is the most common form of kidney failure in hospitalized patients. Any condition that reduces blood flow to the kidney may cause it. The accumulation of these nitrogen-rich compounds in the urine results in a very strong concentrated ammonia-like urine smell. Certain diseases causing liver failure and chronic congestive heart failure of portcaval shunts have been associated with the characteristic odor of dimethyl sulphide [28].

Some infectious diseases are well known for causing the production of diagnostic odors in afflicted patients. The distinctive stench odor of smallpox was very familiar to physicians dealing with this disease during the epidemics of the past. Patients with typhoid fever produce a smell comparable to freshly baked brown bread, whereas individuals with diphtheria have a sweet odor [26]. Diseases such as squamous cell carcinoma and yellow fever release malodorous compounds from the skin [26,29,41]. Patients with yellow fever have an odor that resembles a butcher shop [26,29]. Scrofula, an infectious disease more precisely known as cervical tuberculous lymphadenopathy (tuberculosis of the neck), is caused by the bacteria *Mycobacterium tuberculosis* in adults, but in children it is usually caused by *Mycobacterium scrofulaceum*. Patients with scrofula emit a fermentative odor similar to stale beer. Unpleasant characteristic smells frequently are produced from certain respiratory tract diseases including bronchiectasis, lung abscesses, and ozaena. Osteomyelitis, uraemia, and Fröhlich’s syndrome have been reported to generate characteristic odors [26]. Vincent’s angina, gingivitis, and tonsillitis also may yield smells that are recognizable by the well-trained diagnostician [21,26].

A few common noninfectious diseases and conditions have been associated with characteristic odors. Gout is a chronic form of arthritis that is caused by high uric acid levels in the blood which accumulate and build-up to painful levels in the tissues and joints. Sweat from a gout patient yields a particular sweet odor. By contrast, scurvy victims who have an acute vitamin C (ascorbic acid) deficient are said to produce sweat that has a putrid odor [26]. Starvation victims with ketoacidosis generally have a sweet, acetone-like breath due to elevated levels of ketone bodies in the blood. Ketones are formed when glycogen stores in the liver have run out due to starvation and the body switches to fat metabolism that generates ketones as breakdown products from fat stores for energy production [29].

The ingestion of certain inorganic chemicals, poisons and drugs can produce peculiar breath odors that are indicative of the substances ingested. For example, amphetamine drug abuse can cause a certain type of bad halitosis [21,26]. Human breath with a garlic-like smell may suggest ingestion of penicillin [21], parathione [26], arsenic, tellurium, or selenium [43]; or acute oral poisoning with yellow phosphorus [44]. Cyanide ingestion is indicated by a bitter-almond breath odor [21], whereas a fruity penetrating breath odor is common for chloral hydrate or paraldehyde ingestion [26]. Turpentine poisoning is suggested by the odor of violets in the urine [26], whereas a urine odor that smells like stale water is indicative of acute tubular necrosis, but urine with a concentrated ammonia smell is more likely due to prerenal azotemia [24].

Acute exposure to VOCs through skin contact, ingestion, or inhalation often can be detected in the breath within 6–12 hours after exposure [14]. Some of these have diagnostic odors including diethyl ether, dimethyl sulfoxide, isobutane, tetrachloroethane, trichloroethane, and trichlorotrifluoroethane [45–50]. Disulfiram is typically metabolized and converted into carbon disulfide that is detectable in the breath [51]. Chemical exposure cannot be detected in the breath for many other compounds because they are either decomposed by the liver or eliminated by excretion or exhalation. Schiffman and Williams [52] clarified that unpleasant odors, such as are detected in the breath, are warning signs of chemical exposure and that the exposure to odorants (compounds with odor properties) cause the damage rather than the odor itself. Thus, the odor sensation simply serves as an exposure marker.

### 2.2. Biological Olfaction

Experiments and observations in the area of biological olfaction were necessary and important steps in the evolutionary process toward developing tools for aroma-detection applications in medicine. Before the invention of electronic noses, the olfactory systems of various animals were originally investigated as a means to detect disease in human patients. The domesticated dog was often used due to a keen sense of smell and the ability to discriminate not only faint odors, but also distinguish small differences in odors [8]. Various claims and unofficial reports describing incidences of family pets sensing unusual odors in their human owners were not uncommon. The beginning of interest in testing the hypothesis that dogs might be able to detect diseases in humans probably began in 1989 when Williams and Pembroke described the case of a woman who was encouraged to get a skin lesion sniffed by a dog that showed inordinate interest in a spot on her skin [53]. The lesion proved to be a malignant melanoma after clinical examination. A paper published by Pickel *et al.* [54] provided initial results indicating that the hypothesis suggested by Williams and Pembroke might be valid. Subsequent studies have further demonstrated the feasibility that a dog’s sense of smell can detect certain human cancers in the bladder [55], lung and breast [56], prostate [57], and ovaries [58].

Moser and McCulloch [59] conducted a systemic review of research on canine detection of human cancers and concluded that dogs can detect human cancers with enough sensitivity and specificity to be useful for diagnostic purposes. They thought canine scent detection was a valid and feasible method for cancer detection, but suggested that exhaled breath seemed to be a better non-invasive biological sample than urine for cancer biomarker analysis and early diagnosis of certain cancers. Indeed, more biomarkers of cancer and other diseases have been found in the breath than in any other sample type (see Section 4.1).

Attempts to bridge the gap between biological (living) olfaction systems and artificial olfactory systems (using the electronic nose) are underway in numerous research laboratories throughout the world. Many researchers have taken the approach of “bioinspired” sensor design by attempting to mimic natural biological olfactory systems. Wyszynski and Nakamoto [60] reviewed available gas-sensing technologies and found the Surface Acoustic Wave (SAW) type sensor arrays, including the Quartz Crystal Microbalance (QCM) sensors, were the most suitable choices for artificial olfaction in their opinion, based on sensor design and operating principles. They provided basic information on the structure and principles of various biological and artificial olfactory systems along with methods for the fabrication of biomimetic or bioinspired sensor arrays to develop QCM electronic noses.

### 2.3. The Discovery of Bio-Indicators of Disease

The invention of Gas Chromatography (GC), during the 1950’s and early 1960’s, and GC-linked with Mass Spectrometry (GC-MS) in subsequent years provided the necessary instrumentation to begin to separate and identify individual volatile biomarkers of human diseases. However, one of the biggest early problems with VOC analyses was that it was difficult to capture bioindicator compounds from sample sources of diseased tissues due to the high volatility of gaseous components that allowed escape to the air. An apparatus for sampling volatile skin vapors for GC analysis was devised in 1973 by Distelheim and Dravnieks [61]. This sample-collection apparatus was an early precursor to modern techniques and devices such as chemical trapping, cryogenic trapping, and adsorption trapping by Solid Phase Microextraction (SPME) apparati used to capture VOCs from the gaseous state for chemical analysis. The technique of cryogenic trapping was developed in 1971 by Linus Pauling who constructed a cold trap chilled with liquid nitrogen for human-urine volatiles, and a stainless steel tube cooled in an isopropyl alcohol-dry ice bath for human-breath volatiles to freeze out VOCs that were then revolatilized by heating when injected into the gas chromatograph [62]. Since then, thousands of VOCs have been identified in human breath, but the biochemical significance of most of these compounds is still largely unknown [63,64].

Breath tests were used by physicians from the earliest history of medicine because it was known that the odor of the breath could be altered in individuals with certain diseases [15]. Susruta Samhita, one of the originators of ancient Hindu medicine around 600 BC and the father of modern surgery, recognized the importance of the peculiar smells in the breath and perspiration for identifying many diseases [65]. Some breath aromas have been determined to be highly characteristic of specific diseases. For example, patients with diabetic ketoacidosis smell like rotten apples, mainly due to acetonemia, and chronic renal failure that causes the breath to smell like stale urine [27]. Phillips *et al.* [66] investigated VOCs in breath as possible tumor markers for lung cancer and found two classes of compounds, mainly alkanes and monomethylated alkanes, in lung cancer patients which were absent in healthy individuals. A similar study was conducted by essentially the same research team to investigate the presence of VOCs as markers for breast cancer [67]. Inflammatory lung diseases such as asthma, Chronic Obstructive Pulmonary Disease (COPD), and cystic fibrosis also have been associated with specific volatile markers, including nitric oxide and various hydrocarbons, in exhaled breath [68,69].

Numerous studies have elucidated specific biomarker indicator VOCs, associated with various diseases, using a range of different chemical techniques in addition to GC and GC-MS [70]. Hiroshi *et al.* [71] found trace amounts of two volatile sulfur-containing compounds, methyl mercaptan and dimethyl sulfide, in expired alveolar gas by GC-flame photometric detection (FPD) and GC-flame ionization detector (FID) analysis in 116 total subjects with acute and chronic hepatitis, hepatic cirrhosis, and stomach ulcer and/or biopsy of gastric mucosa. Goldberg [72] used capillary GC-FID and MS to identify VOCs in blood plasma and cerebrospinal fluids of human subjects suffering from hepatic encephalopathy. Of 40 VOCs detected, 21 were identified and only 3-methylbutanal was found to be significantly elevated (*P* < 0.01) in chronic encephalopathics compared to controls. The concentration of this 3-methylbutanal increased with severity of the encephalopathic state.

Ondrula *et al.* [73] utilized a rodent-colitis model to demonstrate that pentane generation and exhalation increased by membrane lipid peroxidation after induction of colonic inflammation by oxygen radicals. They used GC-analysis to show that pentane levels in exhaled air were correlated with damage resulting from oxygen radicals that played a key role in colitis. In this case, the pentane released in expired breath was generated by lipid peroxidation of omega-6 polyunsaturated fatty acid. They found the most deleterious effects of lipid peroxidation on cell membranes were perturbations of membrane fluidity and calcium transport capacity. Formation of free oxygen radicals and subsequent cellular peroxidation of lipids have been implicated in the inflammatory induction of cellular damage seen in many other medical conditions. By contrast, Garner *et al.* [74] analyzed the VOC profiles from feces of colitis subjects using GC-MS to identify possible volatile patterns (specific mixtures) that could be used to diagnose gastrointestinal disease. A total of 297 volatiles were identified with certain compounds (ethanoic, butanoic, pentanoic acids, benzaldehyde, ethanol (acetaldehyde), carbon disulfide, dimethyldisulfide, acetone, 2-butanone, 2,3-butanedione, 6-methyl-5-hepten-2-one, indole, and 4-methylphenol) that were found in all samples. Forty-four compounds were shared by 80% of subjects. When compared to healthy donors, volatile patterns from feces of patients with ulcerative colitis, *Clostridium difficile* and *C. jejuni* infections, were all significantly different.

Research pursuits of bioindicator VOCs in human breath have been the most popular and common approach to disease detection due to the ease with which samples can be noninvasively collected, and the theoretical high probability that many aberrant volatile compounds generated by disease within the body are eventually expelled out of the body through the lungs. A wide variety of different chemical analyses have been used to search for bioindicators of disease in the breath of humans and model mammalian systems. Mottram *et al.* [75] demonstrated the feasibility of an early detection of hyperketonaemia in dairy cows by monitoring concentrations of acetone in the breath and ketones in the milk. Ketosis onset could be detected by GC-MS long before clinical signs (inappetance and a weakened state) became apparent. Likhodii *et al.* [76] found that they could monitor systemic ketosis induced by maintenance of a high-fat, low-carbohydrate ketogenic diet (KD) by measuring breath acetone using GC-FID analysis. They were able to validate the breath-acetone test as a fast, reliable, and noninvasive tool for ketosis assessment suitable for clinical studies of KD for alleviating drug-resistant epilepsy. Lord *et al.* [77] used GC-Ion Mobility Spectrometry (GC-IMS) to investigate acetone and ethanol as biologically important markers of human health and exposure to hazardous VOCs. Karl *et al.* [78] measured human breath VOCs using Proton-Transfer Reaction Mass Spectrometry (PTR-MS) to study the relation between isoprene concentration in the breath and blood cholesterol level. Amann *et al.* [79] utilized PTR-MS for online monitoring of VOC-emission patterns over time during sleep, combined with polysomnography, and in patients suffering from carbohydrate malabsorption. Moser *et al.* [80] employed PTR-MS to determine mass spectrometric characteristics of an “average patient sample” to use as possible reference values for comparisons with future patients. Research of Selected Ion Flow Tube Mass Spectrometry (SIFT-MS), a relatively new technique for real-time quantification of trace VOC gases in breath, has elucidated several potential medical applications by detecting volatile bioindicator compounds [81–89].

Optical Absorption Spectroscopy (OAS) has been used to assess oxidative stress for a range of clinical applications including a wavelength-modulation system that measures ethane in breath [90], and a mid-infrared (IR) high-resolution Tunable Laser Absorption Spectroscopy (TLAS) system to measure exhaled nitric acid and carbon dioxide in human breath [91,92]. Baum *et al.* [93] reported use of Ultraviolet Absorption Spectroscopy (UVAS) to measure acetylene in breath for monitoring cardiac output. Wang *et al.* [94,95] developed laser spectroscopic techniques and a portable breath analyzer based on Cavity Ringdown Spectroscopy (CRS), operating at ultraviolet and near-IR frequencies, to detect acetone in breath.

Colorimetric methods previously were used to measure specific VOCs in human breath, including acetone and hydrogen cyanide [96–98]. Teshima *et al.* [96] applied colorimetric methods to determined acetone in breath by reaction with alkaline salicylaldehyde to form a colored product monitored with GaN-based Light Emitting Diodes (LEDs) by blue-light emission at 465 nm. Corradi *et al.* [99] assayed nitrate (NO_3_^−^) in Exhaled Breath Condensate (EBC) from patients with different inflammatory airway diseases using ion chromatography followed by conductivity measurement using ion chromatography. Nitrate levels were elevated compared to controls in smokers, asthmatics, and patients with Community-Acquired Pneumonia (CAP), but not in patients with Chronic Obstructive Pulmonary Disease (COPD). Balint *et al.* [100] used a chemiluminescence analyzer to discover that 3-nitrotyrosine, a stable end product from the metabolic oxidation of nitric acid, may be a bioindicator of cystic fibrosis (CF). Nitrotyrosine levels in breath condensate were increased significantly in CF patients compared with normal subjects.

Filipiak *et al.* [101] analyzed VOCs released from a lung cancer epithelial cell line, derived from a lung squamous cell carcinoma, by thermal desorption GC-MS. They confirmed that certain VOCs are specifically released or consumed by lung cancer cells indicating the presence of a tumor in the lung. A significant increase in concentrations of four organic compounds (2,3,3-trimethylpentane, 2,3,5-trimethylhexane, 2,4-dimethylheptane and 4-methyloctane) were found in the headspace of cancer-cell cultures compared to controls after incubation for 18 hours. Brunner *et al.* [102] applied multivariate statistical analysis on 42 selected marker VOCs, emitted by *in vitro* cultured cancerous and noncancerous human cell lines and identified with Proton Transfer Reaction Mass Spectrometry (PTR-MS), to clearly distinguish between cancerous and non-cancerous cell lines. Among all of the VOCs identified from headspace volatiles of the cell lines, the most outstanding compound was acetaldehyde that was absent among headspace VOCs of the cancer cell lines, revealing significant consumption by cancerous cells, but not by non-cancerous cells.

### 2.4. Summary of E-Nose Reviews of Specialized Biomedical Applications

We include a brief summary here of some specialized minireviews of electronic-noses that have included various categories of applications pertinent to the biomedical field. In some cases, these reviews provide additional insights and information beyond what is provided in the current review and included additional references in specific aspects of e-nose applications other than the subject categories covered here. Manolis [14] provided an early review of literature describing how breath metabolites have been used for the diagnosis of disease, including neonatal screening, toxicology, and metabolic diseases. Gardner *et al.* [103] assessed research carried out at Warwick University (Coventry, England) to apply an e-nose to the identification of pathogens in cultures and to diagnose diseases from breath samples. Thaler *et al.* [104] evaluated e-nose medical applications such as detecting human pathogens in diseased tissues and monitoring various medical conditions. Lisboa [105] examined the contributions of artificial neural networks (ANN), such as those commonly used in combination with e-noses, to the clinical functions of diagnosis, prognosis and survival analysis within the medical domains of onchology, critical care, and cardiovascular medicine.

Turner and Magan [106] gave some future perspectives on how e-noses are likely to play a significant role in early disease diagnoses, detection of microbial diseases, and in monitoring disease epidemiology. Furthermore, Smith [107] reported on efforts to standardize biomedical terminologies in order to allow for the future integration of new diagnostic technologies, such as e-noses, without creating confusion in biomedical electronic record keeping. Cao and Duan [108] reviewed a range of different diagnostic research methods and techniques for VOC breath analysis including chemical sensors and electronic noses. Probert *et al.* [109] summarized the clinical implications of VOC analysis of various biological emanations including stool, breath and blood samples and their correlation with gastrointestinal and liver diseases.

Mahmoudi [110] cited examples of how major advances in gas sensor technology could enhance the diagnostic power of e-noses to facilitate global surveillance of disease control and management, as well as support of a wide variety of expanding global markets. Voinova [111] summarized current trends and theory in the analysis of physical properties of thin films measured with four different types of mechanically oscillating sensors: (1) the Quartz Crystal Microbalance with Dissipation (QCM-D) monitoring, (2) Surface Acoustic Wave (SAW), (3) Resonators and Magnetoelastic Sensors (MESs), and (4) a new class of acoustic wave mass sensors called Thin-Film Bulk Acoustic Resonators (TFBARs). Qureshi *et al.* [112] described the unique properties and structure of carbon-based electrochemical sensors, including carbon nanotubes (CNT), Diamond-Like Carbon (DLC) films, and diamond film-based sensors. They summarize the most relevant contributions these sensors have provided to the development of e-nose technologies and explain why the properties of CNT sensors are extremely attractive for the development of a diversity of sensor types ranging from amperometric enzyme electrodes to DNA hybridization biosensors. Recently, a carbon-based sensor fast diagnosis method, using non-treated blood assay and amorphous DLC electrodes, was developed for specific detection of human liver diseases, such as chronic hepatitis, cirrhosis, and hepatocellular carcinoma caused by hepatitis B virus (HBV) [113]. Due to these properties, CNT sensors have been utilized in a variety of areas such as electronic, optical, mechanical and biomedical applications [114].

Wilson and Baietto [8] provided a thorough review of theoretical operations, electronic nose detection principles and mechanisms, and examples of commercial e-nose applications developed for diverse industries. This review includes a limited discussion of biomedical applications of electronic noses for human disease diagnoses, and provides some feasibility assessments for new future developments of e-nose technologies in biomedicine. Makino and Yano [115] reviewed published physiological and computational studies of olfaction to understand how the biological olfactory system solves pattern-recognition problems in the real world. Their approach was to address two main issues: (1) the mechanism by which odors may activate the olfactory regions of an animal’s brain, and (2) how the brain solves problems of recognition in the presence of potentially-confusing background odors that may be present in the environment.

Payne [116] wrote an early review of some e-nose characteristics and developments that led to applications in clinical biomechanics. He showed that the ideal e-nose instrument should operate at low cost, be non-invasive (not mechanically penetrate the patient’s skin), utilize nonhazardous forms of energy (low energy sources), operate rapidly, provide precise measurements, be reliable (with little down time), and be safe for both patient and user. Portability (small size and light weight) was added to these characteristics for long-term ambulatory monitoring. Sankaran *et al.* [117] reviewed a wide variety of different electronic instruments and techniques, including electronic noses, which have potential phytopathological and biomedical applications.

## 3. Applications of Electronic Noses in Biomedicine

The earliest studies involving aroma measurements for biomedical applications were attempts to test the efficacy of using the electronic nose to diagnose human diseases caused by microbial pathogens. The initial focus utilized well known pathogenic microorganisms and tested the capability of e-noses to identify microbes through the detection of VOCs they released *in vitro* from bacterial plate cultures or directly from infected tissues [118,119]. In 1997, Gibson *et al.* [120] were able to correctly classify human-pathogenic yeasts and twelve different bacteria at a level of 93.4% using an e-nose with a 16-sensor array and Principal Component Analysis (PCA), a statistical method for comparing headspace volatiles of different samples based on the presence of key (principal) components or organic compounds present in the volatile mixtures. The same year, Chandiok *et al.* [121] used a novel artificial nose to screen patients for bacterial vaginosis infections, and Wilson and Lester [122] demonstrated the feasibility of identifying phytopathogenic microbes based on VOC headspace volatiles released from bacterial and fungal cultures. The Cyranose 320 e-nose was used by Dutta *et al.* [123] to identify three different strains of *Staphylococcus aureus* bacteria responsible for ear, nose, and throat infections in hospital patients. They combined PCA based on three-dimensional scatter plots using Fuzzy C Means (FCM) and a self-organizing map (SOP) network for data analysis. The Cyranose 320 e-nose was capable of identifying three bacterial aroma-subclasses of *S. aureus* strains with 99.69% accuracy. In a similar study, Gardner *et al.* [124] used a metal oxide semi-conducting (MOS) e-nose to predict the growth phase and identity of *Escherichia coli* and *S. aureus*. Head space VOCs were examined using a sensor array with six MOS sensors and aromas were classified by multi-layer perception (MLP) with a back-propagation learning algorithm. MLP identified 100% of unknown *S. aureus* samples and 92% of unknown *E. coli* samples. The growth phases of the bacteria were predicted from analyses of head space samples with an accuracy of 81%.

### 3.1. Major Categories of E-Nose Applications in Healthcare

The early investigations of potential electronic-nose applications for clinical diagnoses and pathology eventually led to the realization that the capability of electronically sampling and instantly analyzing aromatic volatiles in real time directly from patients (or samples taken from patients) could be further exploited to obtain many other types of diagnostic information on medical conditions. The resulting list of potential e-nose applications rapidly expanded into many other medical departments. The conceptual development of new e-nose applications became theoretically feasible in numerous healthcare sectors as new ways were discovered for detecting and monitoring the medical conditions of patients from e-nose analyses of sampled air and VOCs in headspace volatiles. Many of these discoveries resulted from the identification of biological marker (bioindicator) compounds that were correlated with and found to be indicative of specific physiological conditions and diseases. However, the e-nose capability of qualitative chemical detection of specific VOC mixtures also provided uses in many other healthcare-related sectors.

The growth of electronic-nose applications in the healthcare industry has development far beyond the initial uses discovered in disease detection. E-nose applications have been forthcoming and continue to be developed in a wide diversity of healthcare sectors ranging from prognoses and monitoring of various medical conditions to detecting health hazards in the environment, as well as applications in pharmacology, research, medical-waste evaluation, and telemedicine (Table 2). Numerous specific application areas within each healthcare sector have been discovered and realized by the creation of application-specific sensor arrays, odor-recognition algorithms and databases, and by the clever identification of carefully-selected target VOCs for detection.

Wang *et al.* [130] elucidated the application potential of Cell-based Biosensors (CBBs) to detect functional information of biologically active analytes. CBBs have high sensitivity, excellent selectivity, and rapid response time. These sensor characteristics have allowed CBBs to be applied to many fields such as biomedicine, environmental monitoring and pharmaceutical screening. Recent advances in cell-cultured, silicon microfabrication, and genetic technologies promoted dramatic increases in the exploration of CBBs. In related work, Wang *et al.* [131] evaluated the potential of using olfactory and taste cell sensors, a type of cell-based biosensor derived from electronic-nose and electronic-tongue technologies, to mimic living olfactory and taste cells on the surface of sensor chips such as Light-Addressable Potentiometric Sensor (LAPS) to record action potentials representing the odorants and taste in olfactory receptor cells within epithelial cell membranes. LAPS semiconductor chips were used to test the effects of certain stimulative drugs, norepinephrine (NE) and tetrodotoxin (TTX), to elicit action potentials of taste cell and inhibit taste cellular potential, respectively. NE is one of the neurotransmitters within taste tissue which elicits action potential in taste cells. They demonstrated the feasibility that olfactory and taste cell sensors in the form of LAPS chips could be used to study taste-transduction mechanisms to identified ion channels and cell receptors to various drug treatments.

Zhu *et al.* [136] tested the Fox 4,000 MOS e-nose, equipped with 18 metal oxide sensors and a Odorscanner 100 headspace autosampler, for the qualitative and quantitative analysis of flavors in pharmaceutical formulations. Flavor analysis is commonly used in drug development to determine which flavor additives are most effective in masking drug bitterness and distastefulness in order to make oral drug formulations more palatable. Chemometric methodologies including PCA, discriminant factorial analysis (DFA), and partial least squares (PLS) were used for data processing and identification. This e-nose technique was used to identify and qualitatively distinguish between flavorings of placebo drug formulations. The Fox 4,000 e-nose also was capable of discriminating different lots of freshly-prepared and aged flavor samples. These findings confirmed the potential for using e-noses to assay flavor concentration during release-testing of oral-solution formulations, and monitoring flavor shelf-life in different marketed drug containers.

Yao *et al.* [168] compared the performance of aptamer-based and antibody-based quartz crystal microbalance (QCM) biosensors for the detection of immunoglobulin E (IgE) in human serum. Aptamers and antibodies specific to IgE were immobilized on the gold surface of a quartz crystal and frequency shifts of the QCM were measured. The linear range with the antibody was (10–240 μg/L) compared to that of the aptamer (2.5–200 μg/L), but a lower detection limit could be observed in the aptamer-based biosensor. The reproducibility of the two biosensors was comparable, but the aptamers were equivalent or superior to antibodies in terms of specificity and sensitivity. The aptamer receptors could tolerate repeated affine-layer regeneration with minimal loss of sensitivity after ligand binding and recycling of the biosensor.

The application of e-nose sensors as physiological-monitoring devices has provided some unique opportunities to use them for the control of mechanical treatment-delivery devices such as various actuators, valves and pumps. Xu *et al.* [199] developed a novel conducting polymer drug delivery system in which drug release is achieved by electrochemically actuating an array of polymeric valves on a set of drug reservoirs. The valves are bilayer structures, made in the shape of a flap hinged on one side of a valve seat, consisting of thin films of evaporated gold and electrochemically-deposited polypyrrole (PPY). Conducting-polymer (CP) sensors made with PPY have attracted attention because of advantages including easy synthesis and microfabrication, low voltage actuation, wide range of dopant species, high tolerance to electrode materials and electrolytes, good environmental stability, and biocompatibility with physiological environments [211,212].

Phillips *et al.* [192] utilized input data from GC-MS-monitored breath markers of oxidative stress that were input into a specialized computer-based mathematical model to predict the probability of various grades of heart-transplant rejections. The Breath Methylated Alkane Contour (BMAC), a 3-dimensional display of the abundance of C_4_-C_20_ alkanes and monomethylated alkanes, was constructed for every patient. Monitoring with GC-MS is very expensive and time-consuming. Thus, a much cheaper and more rapid (real-time) method of VOC analysis of patient breath air, using electronic noses in tandem with computers loaded with pattern-recognition algorithms, could be achieved once the alkane biomarkers of oxidative stress were known and the corresponding digital electronic aroma signature patterns (EASPs) were inserted as reference databases for comparison with e-nose input data. EASPs could be integrated into the mathematical-prediction models to determine the probabilities of different types of heart-transplant rejections.

### 3.2. Detection of Chemical and Biological Contaminants in Food

The ingestion of food-borne pathogens and associated toxins accounts for a major portion of infections caused by pathogenic microorganisms. Detection of the various human pathogens in foods prior to consumption provides a very effective means of preventing many gastrointestinal diseases. Arora *et al.* [161] summarized a wide diversity of biomolecular and electronics-based methods, including electronic noses, available for the detection of many food-borne pathogens. The high-speed automation of modern food-processing plants has demanded the use of rapid detection systems to catch microbial spoilage in food stuffs. The capability of e-noses to rapidly and noninvasively monitor food-spoilage VOCs in real-time makes these detectors ideally suited for a range of sanitation and safety applications within the food industry, including meat, fish and muscle foods [158]. Currently, the food industry is one of the biggest markets utilizing electronic noses for quality assessment of food production, inspection of food quality, control of food processes, inspection of meats, monitoring fermentation processes, checking for freshness and rancidity, verifying if juice is natural, monitoring food and beverage odors, grading the quality of alcoholic beverages, and inspection of beverage containers and automated flavor control [213]. Recent studies indicate that electronic nose systems have great potential for more effective microbial-contamination control in food-manufacturing processes. Microfabrication methods allow biosensors and other automated electrochemical microarray platforms to be constructed as arrays and incorporated into lab-on-a-chip devices [128,163].

The capability of e-noses to detect toxins and other detrimental microbial metabolites in foods, in addition to microbial pathogens, provides another useful application for food safety and disease prevention [213]. Rahman *et al.* [184] indicated that several novel planar interdigital sensors, based on interdigital electrode structures such as surface acoustic wave (SAW) e-noses, might be used for the detection of marine biotoxins in seafoods. Shellfish toxins and ciguatoxins can cause amnesic shellfish poisoning (ASP), azaspiracid shellfish poisoning (AZP), diarrhoeic shellfish poisoning (DSP), neurotoxic shellfish poisoning (NSP), and paralytic shellfish poisoning (PSP), while ciguatoxins can cause ciguatera fish poisoning (CFP) [214]. A large number of gastrointestinal diseases have been linked to the consumption of raw oysters, clams and mussels. Some of these illnesses are related to the ingestion of domoic acid-contaminated mussels which lead to ASP [214]. The e-nose detection and identification of specific marine biotoxins in foods could prove useful in either prophylactic safety procedures to avoid consumption of contaminated foods or to help diagnose the cause of illness associated with food poisonings.

Electronic noses also have been demonstrated to be capable of detecting fungi and fungal toxins (mycotoxins) in various types of cereal grains. Paolesse *et al.* [215] provided evidence for the feasibility of the electronic nose in the early detection of fungal VOCs in wheat seeds infected with *Penicillium chrysogenum* and *Fusarium verticillioides*. The results of two separate studies indicated the potential of the PEN2 electronic nose with 10 MOS sensors to detect aflatoxins (produced by *Aspergillus flavus*) in corn, useful for screening grain lots for the presence of toxigenic fungi [162,216]. Olsson *et al.* [159] detected and quantified ochratoxin A and deoxynivalenol in barley grains using GC–MS and an electronic nose. Falasconi *et al.* [160] detected toxigenic strains of *Fusarium verticillioides* in corn with an EOS-835 e-nose using 6 MOS sensors.

### 3.3. Detecting Ingestion of Drugs, Alcohol, and Heavy Metals

Several studies have indicated the potential application of electronic noses for detecting the prior ingestion of drugs and alcohol, in cases of substance abuse, and the accidental ingestion of heavy metals such as elemental gaseous mercury [149,150,171]. Alcoholism is a growing problem for all age groups particularly in advanced countries. The number of illnesses associated with excessive drinking continues to rise and the increased rate of alcohol consumption has lead to increases in the incidence of dementia, Alzheimer’s disease, hypertension and cerebrovascular accidents. A recent study by Jin *et al.* [217] was aimed at detecting alcohol concentration (correlated with consumption) and type in expired breath air of elderly people. Beer, Japanese sake, red wine and shochu were detected using a e-nose sensor array with three types of MOS sensors, adopted to examine the VOC components and grade or type of alcohols expired by test subjects. This methodology was used to correctly identify the presence and type of alcoholic substance consumed within 1–3 hours after drinking. Comini *et al.* [150] showed that a MOS e-nose with TiO_2_ films doped with platinum and niobium enhanced sensitivity towards ethanol and methanol. Accidental methanol poisoning is still an occasional problem when alcohol is consumed from homemade stills.

### 3.4. Detecting Hazardous Chemical Presence and Harmful Vapor Exposure

A variety of tasteless invisible gases including CO, CO_2_, SO_2_, NO_2_, radon, and others are a constant threat to human health, particularly in residential homes and in tight living spaces, because these toxic gases can build up in the absence of adequate ventilation especially during cold winter months. Various commercial smoke detectors and gas sensors are available to detect some of these noxious gases, but many other harmful gases that have deleterious effects on human health often go undetected. Much of the hazard associated with the less-common, injurious organic gases such as ammonia, peroxides, and hydrogen cyanide may be easily detected by an e-nose connected to a centralized audible alarm system [142,154]. Indeed, many such systems are being developed for a wide range of harmful gases and aerosols [139].

Another group of harmful gaseous substances are organic solvents that may be present in ambient air within confined air spaces of hospitals and various industrial working environments. These substances volatize from many sources including certain plastics, styrofoam and other insulative products, paints and varnishes, and open solvent containers. Many e-nose methods have been developed for the detection of exposures to toxic and injurious organic solvents such as methanol [141,143], ethanol [144,147], chloroform [151], and other hazardous organic gases [146,152].

### 3.5. Detecting Microbes in Potable Water and the Environment

Potable water resources for drinking and numerous ablutive medical procedures are vulnerable to contamination by microbial and chemical agents in the environment. The detection of harmful substances in potable water sources is essential to human health. Several research studies demonstrate useful e-nose methods for the detection of human pathogens in a variety of environment sources. Bastos and Magan [172] used a Bloodhound BH-114 conducting polymer e-nose to detect pathogens in seafood and potable water. Similar methods were used to detect microbes in soil and water samples [173]. Chang-Yen *et al.* [205] utilized an optical sensor e-nose to quantify oxygen concentration for a variety of applications, including, environmental monitoring, biological respiration studies, and cell culture. Oxygen concentration is often indicative of anaerobic conditions in water sources with microbial contamination.

Coliform bacteria are common microbial contaminants of potable water supplies that are located in close proximity to sewer lines and sewer discharges into open water supplies and near shallow wells. McEntegart *et al.* [218] provided evidence that the Moses II e-nose with 8 MOS, 8 QMB, and 4 AGS sensors could be used to detect and discriminate between different types of coliform bacteria in contaminated samples using pattern recognition of VOCs produced by these microbes during growth. This method would be useful to identify the presence of coliform bacteria in water processed from major water sources such as open reservoirs often used as drinking water sources for many local municipalities and hospitals.

### 3.6. Medical Instruments and Waste Evaluation

Several applications of e-noses are proposed here for checking the sanitation condition of hospital instruments, equipment, needles and other biomedical wastes. For example, medical instruments and equipment to be used in operations could be checked for contamination by chemicals or microbes, via e-nose sniffing for diagnostic VOCs, immediately prior in a medical operation or procedure to assure that these critical instruments and equipment have been adequately cleaned and sanitized to avoid adverse effects to the patient. Likewise, the presence of hazardous chemicals or contagious microbes in biomedical wastes could be tested to make sure such wastes have been properly decontaminated or neutralized by autoclaving or other forms of sanitation measures before these medical wastes are finally disposed of in sanitary landfills [7,8]. E-noses also could be used to check the gas mixtures of anesthetizing agents prior to application to avoid complications caused by altered or improper mixtures. All of these pre-emptive safety checks could be quite useful in avoiding unnecessary mistakes and complications due to inadequate preparation of materials and equipment prior to medical procedures. These measures could potentially save lives and reduce the incidences of malpractice associated with negligence resulting from inadequate preventative safety measures or accidents.

### 3.7. E-Nose Applications in Remote Telemedicine

The potential uses of portable electronic noses for medical applications in remote locations (away from hospitals) are enormous. Combining advanced telecommunication devices such as teleconferencing and videoconferencing with the diagnostic capabilities of e-noses could vastly improve the quality of healthcare in remote locations of the world. Practitioners trained in e-nose operations could be in direct contact with doctors and specialists in hospitals to determine the health condition of ailing patients in the field and administer appropriate treatments. E-nose results could be uplinked via satellite, transmitted through fiber-optic phone lines or cable, then output onto the computer screens of advising doctors in the hospital [219]. These uses of rapid real-time e-nose analytical methods in combination with advanced telecommunication devices are well within the capabilities of current technologies.

Systems for the transmission of medical information from the field have already been developed and used in remote locations in Africa [206]. One such system was developed with the capability of discriminating patients with diabetic mellitus, gastric tuberculosis and other diseases that cause halitosis. Jamal *et al.* [220] proposed a real-time recognition method using a handheld OMX-GR e-nose with an Artificial Neural Network (ANN) analysis system trained with a back-propagation algorithm. The identified odors were transmitted electronically from the electronic nose itself (at the site of the patient) to doctors or other trained medical personnel at a different location. They also proposed a new method for regenerating the transmitted olfactory information. The combination of localized diagnosis using an e-nose and data transmission methods provides a means of extending the effective range within which medical doctors can provide services to remote locations of the world.

## 4. Disease Diagnosis Using the Electronic Nose

A major challenge for modern medicine is to achieve effective disease diagnoses through early detections of pathogenesis or disease conditions to facilitate rapid corrective or curative treatments, but at the same time reduce the invasiveness of diagnostic treatments [8]. Hitherto, the most common means of diagnosing most pathological conditions was to conduct chemical analysis of human samples, such as breath, blood, urine, sweat, or other bodily fluids. Thus, current clinical diagnoses, based on the metabolic profile concept, are largely limited to investigations and examination of human fluids [221,222]. Pathogenic microbial species produce a wide range of VOCs. The diagnostic potential of pathogen identification through analysis of microbial metabolites was first recognized and considered theoretically possible in the early 1960s [223]. Use of traditional VOC chemical analyzers such as GC-MS has always been very expensive and too time consuming to allow rapid diagnoses. Differences in the aromas of diseased *vs.* healthy human tissues have provided means for diagnosing human pathogenesis and other abnormal health conditions. This correlation has been supported by studies using the keen olfactory abilities of well trained dogs whose sense of smell is one million times greater than the human nose. Canine olfactory systems have the ability to detect a variety of different diseases including melanomas [54], bladder cancer [55], and lung and breast cancers [56]. This research provided theoretical evidence of the potential for using electronic noses in disease diagnosis.

The range of demonstrated applications of electronic noses in the field of clinical medicine have included the capabilities of monitoring microbial metabolites released from superficial wounds [224], detecting volatiles associated with upper-respiratory tract infections (in rhinology) [225], diagnosing disease associated with alterations of VOCs in human breath (such as diabetes) [226], and distinguishing cerebrospinal fluid from serum [227,228]. Many other promising diagnostic applications developed for the electronic nose have included the detection of ventilator-associated pneumonia [229–231], identification of bacterial pathogens [232,233], early screening for the presence of various types of cancers [66,234], breath analyses for detection of lung and other organ-related diseases [235], toxin exposure [236], and radon ingestion [237]. The following subsections summarize in greater detail some of the more promising and useful e-nose applications developed, or under development, in different areas of human and in some cases animal (veterinary) disease diagnosis.

### 4.1. E-Nose Detection of Chemical Bio-Indicators of Disease

The use of traditional analytical instruments such as GC-MS to detect biomarkers of disease has not become routine for clinical applications largely due to the high cost, high complexity, slow results, and difficulty of identifying diagnostic chemical species among a myriad of compounds present in human test samples. The need for chemical sensors and instruments containing sensor arrays has become increasingly necessary to identify VOCs as simple or complex sample mixtures for disease diagnoses. The world-wide demand for intelligent, fast and inexpensive measurement systems for clinical diagnosis is increasing. Electronic noses, used for characterizing and identifying complex vapors and aromas, are useful for detections of microbial contamination or diagnosis of infections. Consequently, the electronic nose was the logical instrument of choice for disease diagnoses due to the capability of identifying complex mixtures of VOCs (as a whole) in addition to individual compounds (in some cases) within sampled air using pattern-recognition algorithms. Once VOC biomarkers of disease have been identified by tradition chemical means, known pure samples of biomarker compounds and mixtures can be prepared and used to develop corresponding pattern-recognition libraries for electronic noses that allow the identification of these specific compounds and mixtures associated with particular diseases. The collective output from the e-nose sensor array, produced in response to a specific group of VOCs (present in the sample air mixture) that come in contact with individual sensors in the sensor array, produces a unique Electronic Aroma Signature Pattern (EASP) that is unique to that sample mixture [7]. Even changes in the molar ratios of individual component VOCs present in a sample mixture give rise to a different e-nose output pattern. Thus, subtle changes in physiological processes and metabolic pathways can generate different VOC mixtures and correspondingly different EASPs.

Most of the VOC biomarkers of disease were originally isolated and identified with traditional analytical instruments and methods due to the complexity of analyses required to reveal their chemical structures. In some cases, a single biomarker compound has been associated with certain diseases and metabolic (physiological) disorders, but in other cases a mixture of aberrant compounds has been found to be associated with specific diseases. A summary of some important biomarker compounds, associated with specific diseases and metabolic disorders, which could be potentially identified with e-nose technologies are presented in Table 3.

Some of the most promising clinical applications being developed using electronic-nose technologies have been for the early detection and diagnosis of diseases that can be detected by the presence of aberrant VOCs in human breath or expired air. In many cases, disease-associated VOCs originate deep within the body in various diseased organs and tissues, but these compounds are circulated in the bloodstream and eventually are released from the body mainly through the lungs, but also through the urine, feces, and sweat.

Hundreds of VOCs have been identified in human breath [62], and some of these compounds are associated with specific diseases [14,238–239]. As new biomarkers of disease are discovered, use of the electronic nose for detection of these biomarker VOCs should become increasingly useful for clinical disease diagnoses. Some specific biomarkers that have been identified in association with certain human diseases by various research studies are summarized in the following discussion.

Higher levels of certain alkanes (hexane and methylpentane) have been reported in the breath of patients affected by lung cancer compared to patients with healthy lungs [64,66,293]. Di Natale *et al.* [283] conducted initial studies to evaluate the feasibility of the e-nose in detecting these cancer-marker compounds. They collected breath samples from 35 individuals affected by lung cancer, 9 that just had surgical therapy and 18 controls. Two additional individuals were measured before and after surgical therapy. The electronic nose successfully detected 100% of lung cancer patients, 94% of healthy controls and 44% of post-surgery patients. In this study, an e-nose containing eight quartz microbalance (QMB) gas sensors, coated with different metalloporphyrins, was used. Yu *et al.* [294] and Chen *et al.* [295] confirmed that the QMB e-nose could successfully identify eleven diagnostic VOCs that were validated as bioindicators or chemical markers of lung cancer.

Thorough investigations of e-nose detections of urinary trait infections have been done [258,270]. Aathithan *et al.* [258] conducted e-nose analysis of 534 clinical urine specimens of which 21% had significant bacteriuria indications. Electronic nose diagnoses were done at significantly lower costs and with greater sensitivity (83.5%) and specificity (87.5%) compared with conventional cultural counts. Pavlou *et al.* [296] proposed the use of the electronic nose as a potential diagnostic tool for patients afflicted with various kidney diseases. They were able to distinguish traces of blood in urine samples and obtain rapid identifications of *E. coli*, *Proteus* spp. and *Staphylococcus* spp. infections at very high levels of confidence. Lin *et al.* [297] developed an e-nose application for uremia diagnosis.

Electronic-nose applications for diagnosis of diseases using samples taken externally from patients on body surfaces or from cultures of swabs taken from external orifices also have been developed. Boilot *et al.* [298] used a commercially-available electronic nose to classify six bacteria responsible for eye infections and ENT (ear, nose and throat) disease with an accuracy of 97.3% and 97.6% respectively. They effectively discriminated between pure laboratory cultures containing fixed volumes of bacteria in suspension and blood agar plates used to culture samples collected from diseased patients. Di Natale *et al.* [299] developed e-nose methods for the identification of diseases by odor analysis of VOCs from the human skin.

Headspace analysis techniques have been developed to directly sample volatile metabolites produced by microbes in culture. These techniques involved sampling the culture headspace directly for aliphatic acids [11,300], amines [11], and sulfides of *Proteus* spp. [301], or via use of volatile-concentration methods [302–304]. Headspace analysis has been applied to samples of human body fluids including saliva [305], urine [306,307], and blood serum [308]. Anaerobic microbes generally are more limited in their production of volatile metabolic products compared to aerobic organisms that generate a wide variety of unique metabolites due to their ability to oxidize reduced substrates to various oxidation states [309–311]. Similarly, bacteria tend to produce a greater variety of oxidized volatile substrates than fungi which generally do not oxidize their substrates (as energy sources) to the same extent as indicated by large differences in e-nose sensory array outputs produced from headspace volatiles of these two groups of microbes [7].

Various odors and associated biomarker VOCs have been identified from cultures of a number of parasitic and pathogenic microbes, and in some cases, unique chemicals or chemical groups have been identified (Table 4). A wide diversity of different volatile secondary metabolites is produced as useful biomarkers even by different species within individual pathogenic bacterial genera. For example, pyrazines are produced by *Pseudomonas taetrolens* and *Pseudomonas perolens* [312,313], 2-aminoacetophenone and dimethylsulfides by *Pseudomonas putida* and *Pseudomonas aeruginosa* [311,314], and isopentanol by *Pseudomonas putrefaciens* and *Pseudomonas maltophilia* [311]; esters, sulphur-containing compounds, and I-undecene by *Pseudomonas* species growing on beef products [315]. Similarly, *Proteus* species also produce a variety of different VOC biomarkers by various metabolic pathways. Biomarkers of *Proteus* species range from complex aldehydes and sulfides to a variety of sulfur-containing compounds (dimethyl sulphide, methyl mercaptan), trimethylamine, acetates, long-chain alcohols, and ketones [301,316,317].

The production of the same identical biomarker VOCs can be indicative of several different diseases cause by totally different bacterial species. Certain gastrointestinal and gastroesophageal diseases caused by *Enterococcus* species, *Escherichia coli,* and *Helicobacter pylori* are all known to produce terpenes, trimethylamine, and ketone bioindicator compounds [244]. *Vibrio cholerae* that causes cholera and *Proteus* species that cause gastroenteritis are both producers of the bioindicator dimethyl disulphide. The bioindicator compound I-undecene is produced in common by *Staphalococcus aureus* that causes staph infections and *Proteus mirabilis* that causes the majority of *Proteus* infections associated with gastrointestinal diseases [288,317,318].

Recently, the use of aroma analysis in clinical diagnosis has been rediscovered due to major advances in odor-sensing technology and artificial intelligence. The use of VOC biomarkers in disease diagnosis will likely become increasingly important in the future as more information becomes available on new biomarker indicator compounds associated with specific diseases, and as biomarker detection with electronic noses becomes routine in clinical practice. Increasing worldwide awareness that bionics and artificial intelligence will play an important role in microbial analysis has prompted the development of intelligent data-warehouse systems consisting of odor-generation mechanisms for rapid aroma volatile delivery and recovery (from human samples) coupled with classifier systems based on neural networks and genetic algorithms [318,319]. Microbiological warehouse environment systems adopt the concept of fusion of multiple classifiers dedicated to specific feature parameters or applications. Such data-warehouse systems should ultimately produce a worldwide database that can be accessed and utilized by researchers and developers of medical aroma-based disease-diagnostic instruments, such as electronic noses, to generate reference libraries that may be used to detect VOC biomarkers for the identification of specific diseases. In this way, application-specific e-nose systems with limited recognition libraries may be developed for a fairly narrow range of diseases to limit instrument costs and increase portability through reducing the number of sensors (in the sensor array) to the minimum needed for effective discriminations [7,8].

### 4.2. E-Nose Detection of Nosocomial Diseases

Nosocomial infections are microbial infections that result from treatment in a hospital or a healthcare service unit. Infections are considered nosocomial if they first appear 48 hours or more after hospital admission or within 30 days after discharge. This type of infection is also known as hospital-acquired infection or healthcare-associated infection. The Centers for Disease Control (CDC) estimate that roughly 1.7 million hospital-associated bacterial infections of all types cause or contribute to 99,000 deaths annually [322]. Nosocomial infections are commonly transmitted when hospital officials become complacent or personnel do not practice correct hygiene consistently [323]. The increased use of outpatient treatments has resulted in hospital patients that generally are more ill and have more weakened immune systems than in the past.

Methicillin-resistant *Staphylococcus aureus* (MRSA) and methicillin-susceptible *S. aureus* (MSSA) are two hospital-linked staph strains that can cause mild to severe infections of bones, lungs, heart and blood stream. Some species of *Staphylococcus*, such as *S. epidermis* and *S. saprophyticus*, are known as coagulase-negative staphylococci (C-NS) that are among the most commonly isolated bacteria in clinical microbiology laboratories and can cause infections to immunocompromised patients. Lai *et al.* [232] analyzed swabs of *Staphylococcus aureus*, *Streptococcus pneumonia*, *Haemophylus influenza* and *Pseudomonas aeruginosa*, all common upper respiratory bacterial pathogens, using the Cyranose 320 e-nose. Discrimination between the sample types was performed using pairwise comparisons with Mahlanobis distance (MD). The electronic nose could easily distinguish between the four bacterial species and controls as indicated by MD values of 3 or greater for pairwise comparisons in all possible combinations.

A relatively small group of microbial pathogens are opportunistic generalists that possess pathogenic determinants (*i.e.*, chemical or genetic components that are responsible for disease-causing mechanisms) that enable them with the capability of causing disease in a wider group of unrelated multicellular organisms. Pathogenic determinants produced by virulent bacteria can be such things as toxins, destructive enzyme and protein systems, polysaccharide slime, and plasmids that are the actual integral molecular machinery actively involved in causing or generating the disease process (pathogenesis) and immune responses. As a consequence, these pathogenic microbes are capable of causing diseases in both plants and animals, particularly humans in debilitated states such as patients receiving treatment or recovering from illnesses in the hospital setting. Nosocomial diseases can occur as a result of infection of patients by phytopathogenic microbes derived from infected plants. The capability of certain plant pathogenic microbes to “jump” from infected plants or plant materials to debilitated human subjects has long been known as one of the potential exposure hazards prevalent in the hospital environment. Compromised patients can become exposed to these microorganisms through infected plants, particularly flowering plants that are brought into the hospital and given as gifts to patients. The giving of plants or plant materials has since been strongly discouraged or forbidden in many modern hospitals to minimize the risks of nosocomial secondary infections that can potentially complicate or prolong the patient-recovery phase associated with the original illness. The strongly opportunistic capabilities of these select microbes to cause pathogenesis in unrelated higher organisms has been an area of fascination and a subject of research that particularly lends well to e-nose applications due to the commonality of mechanisms responsible for disease development in all multicellular hosts, whether plants, animals, or humans. A list of these opportunistic nosocomial microbes, capable of infecting both plants and humans, that potentially could be detected using e-nose technologies are presented in Table 5. Although this list is not comprehensive, it represents some of the more common microbial pathogens present in the hospital environment, particularly prevalent on plant surfaces or as airborne spores that are brought into the vicinity of susceptible human subjects.

Nosocomial diseases of humans, caused by opportunistic bacterial and fungal plant pathogens, are possible largely due to the existence of common virulence factors for pathogenicity and disease mechanisms that operate equally well against both plant and human hosts [372]. These microbes take advantage of accidental or incidental entries into the human body that often occur due to skin contact with plant parts. For example, *Pantoea agglomerans*-infections usually occur following tramal skin penetration by vegetative thorns, spines, or prickles that have surfaces contaminated with a resident population of this pathogen [330]. Three other bacterial genera (*Burkholderia*, *Pseudomonas* and *Ralstonia*) that frequent surfaces of plant roots and foliage also are capable of causing opportunistic infections in vulnerable individuals, particularly patients with cystic fibrosis (CF) [336]. Thus, these bacteria also are opportunistic in taking advantage of underlying physiological conditions, compromised immunities, and debilitating diseases, creating significant problems in clinical settings due to their widespread cause of nosocomial infections.

The *Burkholderia cepacia* complex (BCC) is a group of bacteria that comprises at least nine distinct *Burkholderia* species [325]. The type species was originally identified as a plant pathogen by W. H. Burkholder in 1950, when it was identified as the causative agent of onion soft rot disease called sour skin [327]. BCC bacteria are ubiquitous in the environment and important opportunistic pathogens that cause lung infections in CF patients, resulting in asymptomatic carriage, chronic infection or ‘cepacia syndrome’, characterized by rapid decline in lung function. Like other opportunistic pathogens such as *Pseudomonas aeruginosa*, BCC strains do not normally infect healthy individuals, but only those whose immune systems are compromised [373]. CF patients are particularly susceptible to BCC lung infections that have a significant impact on morbidity and mortality [373,374]. BCC bacteria are frequently cultured in the blood due to the systemic nature of the infections that often lead to sepsis and pneumonia, the second most common cause of death in Chronic Granulomatous Disease (CGD) patients [375,376]. Within the past 20 years, BCC bacteria have emerged as highly problematic human pathogens in CF and immunocompromised individuals, and the incidence of BCC infections have increased in these patient groups [377,378]. This increase may be due to increasing numbers of immunocompromised patients, increased population age of these patients [379], more accurate identification of BCC microbes [380,381], social and behavioral changes allowing close contact between patients [382], and increased use of antibiotics to which BCC bacteria are resistant [383].

Many bacterial plant pathogens in the genus *Pseudomonas* have long been known to cause human diseases. Patients who are in intensive care units for extended periods of time are most at risk for contracting a disease caused by a *P. aeruginosa* infection, especially burn victims and those with cancer or cystic fibrosis. The major nosocomial pathogens that attack debilitated patients in intensive care units include *Pseudomonas aeruginosa*, *Acinetobacter baumannii*, *Stenotrophomonas maltophilia* and *Burkholderia cepacia. Pseudomonas cepacia* was transferred to a new genus, *Burkholderia* in 1992 [384].

*Ralstonia* is a relatively new genus that includes former members of *Burkholderia spp.* (*Burkholderia pickettii* and *Burkholderia solanacearum*) commonly found in water and soil in the environment. *Ralstonia pickettii* has been isolated from a wide variety of clinical specimens including blood, urine and cerebrospinal fluid [385]. Although *Ralstonia pickettii* is regarded to be of minor clinical significance due to its low virulence, a wide range of invasive and severe infections have been reported indicating that this microbe may be a more widespread and serious pathogen than previously thought [386]. Infections include bacteremia and septicemia caused by contaminated solutions, e.g., distilled water, water for injection and aqueous chlorhexidine solutions [386]. It also has been identified in ultrapure water systems of industry [387], the space shuttle [388], and laboratories [389]. Cases of pseudobacteremia and unusual infections have been associated with *R. pickettii* with some being very invasive and severe such as meningitis, septic arthritis and osteomyelitis that have resulted in a significant number of clinical deaths [386].

*Acinetobacter baumannii* is an important pathogen that is increasingly recognized as the cause of severe infections in compromised hospitalized patients [390,391]. Deadly cases of community-acquired pneumonia have been reported among immune-compromised patients [392,393]. These infections are difficult to treat due to the expanding antibiotic resistance of clinical strains [394,395]. *Acinetobacter baumannii* is a metabolically versatile pathogen, yet little is known about the genes and factors involved in its basic physiology and virulence properties. Some evidence indicates that the ability of *A. baumannii* to produce and secrete certain proteins for biofilm production may be involved in pathogenesis [396].

Detection of nosocomial bacteria and diseases using electronic noses can be achieved with the same methods utilized for their detection as microbial plant pathogens, even though not all nosocomial pathogens are plant pathogens. Wilson *et al.* [7,122] pioneered the application of the electronic nose for the detection and identification of plant pathogenic (phytopathogenic) microbes, including bacterial and fungal plant pathogens, the diseases they cause in multicellular higher plants [7,122,397], wood decay fungi and incipient wood decay [7,398], and the identification of plants and woody plant hosts by the unique Electronic Aroma Signature Patterns (EASPs) produced by various e-nose sensor arrays in response to headspace volatiles [399]. These e-nose applications for the field of plant pathology developed out of the realization that all pathogenic microbial species produce a very specific combination or mixture of volatile metabolites that are products of various specialized metabolic (both anabolic and catabolic) pathways involved in biosynthesis and the production of energy for cellular activities. The typical sensor array in an electronic nose can detect and distinguish between different specific mixtures of volatile metabolites, produced by different microbial pathogens, as unique electronic digital fingerprints that identify a specific volatile mixture as being produced only by one particular microbial species. The diagnostic recognition of these very specific mixtures of volatiles of particular microbes is possible by using application-specific reference libraries (recognition files), created from samples of known pathogenic microbes, allowing the identification of the causal agent(s) of unknown diseases based on VOC headspace volatiles released from diseased tissues. The response-specificity of the sensory output from a sensor array is so large that differences in response patterns are produced from mixtures containing exactly the same components (chemical species) when only the molar ratio of components in the mixture is changed [7]. Thus, differences in EASPs result even when there are changes in the relative amounts of VOCs present in headspace volatiles. This is important because many microbes utilize many of the same metabolic pathways, but generate different ratios of metabolic products due to slight changes or substitutions in the sequences of chemical reactions that occur within a metabolic pathway or to different proportions of different pathways utilized in their metabolism. The result of different molar ratios of metabolites, generated by variable metabolic pathways in operation in different microbial strains, is accounted for by different proportions of metabolic products produced as a result of unique pathway usage that is governed by gene-regulation (genetic) processes.

Nosocomial mycoses caused by *Fusarium* species are particularly common secondary infections associated with debilitated patients in the hospital setting. Fusaria are facultative fungal saprophytes that survive on overwintering plant debris, remaining after the harvesting of field crops, or as resistant spores (chlamydospores) in the soils of agricultural fields. For example, these saprobic fungi are commonly found on overwintering cereal crops [400]. The spores of these fungi become airborne from soil particles or plant surfaces when fields are cultivated in the spring or crops are harvested at the end of the season. Air-borne spores gain access to the hospital environment primarily through hospital air exchanges with the outside via air entry/exit points. Inoculum is dispersed through air vents. *Fusarium* species often cause sapronosis-type diseases that are defined generally as diseases caused by saprobes in patients or subjects with impaired resistance [401]. *Fusarium* skin infections often have been associated with burned human tissues [402,403], facial granulomas [404], skin lesions [360,361], and eye infections or keratosis [405,406]. Once *Fusarium* inoculum is in the body, it can disseminate to other areas of the body through the circulatory system and infect internal organs [358,407–409]. Infections due to *Fusarium* species also have been associated with patients having osteomyelitis [410], catheter-associated fungemia in lymphocytic lymphoma patients [409], myasthenic syndrome associated with aplastic anemia [407], and renal and bone marrow transplants [411,412].

Electronic noses may be used to detect either *Fusarium*-specific VOC metabolites or toxins, produced by the somatic hyphae of these fungi in plant residues, or released in isolated pure cultures of these fungi [7]. The detection of *Fusarium* mycoses in human burn wounds and infected organs is made possible by the identification of abnormal fungal metabolites released into infected human tissues. The careful selection of sensor types (within the sensor array of the e-nose) which are sensitive to the specific chemical groups of metabolic VOCs produced by *Fusarium* species within affected tissues, provides optimal means for detection of *Fusarium* infections and allows for a high level of selectivity required of application-specific detection methods. This approach assures that e-nose detections are both cheap and effective for real-time diagnoses. Aroma recognition libraries utilized with pattern-recognition algorithms, developed specifically for *Fusarium* VOC metabolites or toxins, would provide the greatest specificity of detection. *Fusarium* species produce a wide range of important toxins in plant residues, some of which are carcinogens, such as beauvericin [413], fumonisins, [414,415], trichothecenes [416], as well as deoxynivalenol, T-2 toxin, HT-2 toxin, and T-2 tetraol [417]. *Fusarium*-specific toxins may serve as effective targets for e-nose detection if similar toxic analogs are constitutively produced in human tissues.

## 5. Electronic-Nose Instruments Used for Biomedical Applications

Electronic-nose applications developed for the biomedical field are growing at a rapid rate due to increasing demand for simple and effective solutions to the numerous problems and diagnostic needs of modern medicine. The strong inclination of private industrial biotechnology firms to shift their research and development (R&D) departments toward development of e-nose technologies for the medical industry is related to the higher cost of detection-instruments needed for disease diagnoses, increasing demand for specialized detection instruments at large numbers of medical and research facilities, the greater availability of funding for instrument purchases, and the increased visibility of biomedical needs [8]. The shift in R&D activities of commercial companies to development of electronic noses also has occurred in response to social, economic, and profit-motivated factors. Some instrument-development companies have shifted their entire R&D programs toward e-nose biomedical applications. Electronic-nose instruments developed for diagnostic medical applications often are more specialized, higher priced, and more lucrative for commercial development. With numerous diverse and specialized applications possible, there are many motivations for e-nose producers to focus their R&D efforts in the field of diagnostic medical-instrumentation development.

A wide diversity of biomedical applications has been developed from commercially-available electronic noses since 1998 (Table 6). These applications are many and varied and range from specific diagnostic detections of microbes, hazardous chemicals, toxins and various types of infection, to drug formulation and development. The number of sensors used in the arrays of these e-noses varies widely.

The development of various biomedical applications using electronic-nose technologies has involved more than a dozen different commercially-available e-nose instruments. Hitherto, the vast majority of e-nose instruments employed for medical applications, including Amperometric Gas Sensors (AGS), Conducting Polymers (CP), Metal-Oxide Semiconductors (MOS), Quartz Microbalance (QMB), and Surface Acoustic Wave (SAW) sensors, represent a relatively small number of e-nose technology types that are available. Most biomedical e-nose applications have involved the use of MOS sensor arrays containing a variable number of sensors. The utilization of MOS sensors is somewhat surprising given that metal-oxide semiconductor sensors generally must operate at high temperatures and require high power consumption for detection. These requirements increase operating costs and greatly limit portability that is required for mobile medical services. MOS sensors also have limited VOC-sensing range with the capability of detecting only low molecular weight compounds. Furthermore, MOS sensors are sensitive to humidity, susceptible to sulfur and weak acid poisoning, and have limited sensor coatings [8]. By contrast, sensor arrays consisting of CP, QMB, SAW and optical sensors are probably better suited for most biomedical applications due to the capabilities of detecting a very wide range of VOCs (in terms of both chemical-class diversity and molecular-weight range) and the more preferred characteristics of high sensitivity and precision, good response and recovery times, small portable sizes, and relatively low purchase and operating costs. Many types of biosensors also are being developed for medical applications and will likely provide another very fruitful area for diagnostic methods development.

### 5.1. Diabetes

Great progress has been achieved in the use of e-nose devices to diagnose and monitor diabetes in clinical patients. Ping *et al.* [226] developed a self-made MOS e-nose for the diagnosis of diabetes. Their analyses were performed by a prototype with thin-film sensors coated with SnO_2_, having high selectivity for acetone, the most important chemical marker detected in the exhaled breath of diabetic patients. In a more recent study, Guo *et al.* [429] classified subjects with renal failure before and after hemodialysis using breath samples captured and analyzed with an electronic-nose gas sensor. They successfully applied a similar MOS e-nose prototype system to distinguish between 14 healthy and 18 diabetic subjects suffering from diabetes, renal disease and airway inflammation based on analyses of breath odor. Cluster analysis indicated that diabetic and non-diabetic subjects sampled before a meal were diagnosed correctly, whereas some controls were mistaken for diabetics due to the rise in acetone concentration associated with hunger. One hour after a meal, 100% of diabetics were discriminated from non-diabetics using the e-nose breath tests.

### 5.2. Nasal Infections

Subjects affected by chronic nasal or paranasal infections often are characterized by peculiar odors in their nasal out-breaths that are usually associated with bacterial or fungal colonization of the sinuses. A first study aimed at examining nasal out-breath samples from patients with chronic rhinosinusitis (CRS) was done using an electronic nose developed by Mohamed *et al.* [430] in 2003. This e-nose correctly classified diseased and healthy controls in 80% of the samples. They employed ANN analysis to correctly classified 60% of the VOC patterns of both groups. In a follow-up study conducted by the same research group, Bruno *et al.* [431] employed a new and more sophisticated electronic nose (ZNose^™^) based on both gas-chromatography and Surface Acoustic Wave (SAW) sensor technology capable of detecting pictograms of VOCs in less than 10 seconds. In pathologic subjects, six odorous components ranging from C6 to C14 were found useful in the diagnosis of chronic rhinosinusitis.

### 5.3. Urogenital Infections

The diagnostic criteria of urogenital infections are based on macroscopic and microscopic characteristics of urine together with chemical and/or physical analyses of the blood or urogenital discharges. In particular, the diagnosis of bacterial vaginosis, one of the most common causes of vaginal infection, is performed by the appearance and pH of vaginal discharge and the emission of amine odor after the addition of KOH. Chandiok *et al.* [121], conducted a study to test the feasibility of an electronic nose to diagnose bacterial vaginosis with urine samples taken from 68 women. They found 94% of known positive cases were recognized as positive by the e-nose using a trained ANN. Approximately 43% were negative based on clinical criteria and 33% were recognized as negative and 23.3% as positive by the e-nose. The positive predictive value for the combined test (e-nose plus clinical assessment) was 61%.

The presence of blood and VOCs in urine samples of children affected by kidney diseases was studied by Di Natale *et al.* [270] in 1999. A Quartz Microbalance (QMB) electronic nose prototype was used to evaluate the pH and specific weight of urine samples. The presence of microorganisms in a large number of human urine samples was analyzed by Aathithan *et al.* [258] in 2001 using an e-nose. They employed the Osmetech Microbial Analyzer, a headspace analyzer with an array of four Conducting Polymer (CP) gas sensors, to detect bacteriuria in 534 clinical urine specimens. The results showed an accuracy of 72% and a specificity of 90% in samples with a microbial concentration of ≥10^4^ colony forming units (CFU) per milliliter, and an accuracy of 83% and a specificity of 88% when the microbial concentration was ≥10^5^ CFU/mL.

Kodogiannis *et al.* [292,432] employed the Bloodhound BH-114 e-nose, based on 14 CP gas sensors, for the early diagnosis of infections in the urinary tract of 45 suspected cases. The study demonstrated that this e-nose, combined with advanced learning-based processing tools, was able to identify specific bacterial pathogens with high speed and accuracy. Witt *et al.* [433] used an e-nose with CPA and a quadratic discriminant algorithm to analyze the skin volatiles at the skin surface of patients with liver cirrhosis and addiction to alcohol. They found sweat volatile gases are changed because of the liver dysfunction and remaining alcoholics and alcoholic decomposition products within the blood. These diseases lead to changes in metabolic balance. They developed a special applicator to collect and analyze the transpired dermal gases directly on the skin surface. They showed in a pilot study on 25 patients that an electronic nose is able to detect changes in the human body odor and to discriminate between healthy subjects (controls), patients with liver cirrhosis and patients addicted to alcohol.

## 6. Biomedical Research Developments for Electronic Noses

Electronic-nose systems have been designed specifically to be used for numerous applications in many different biomedical applications. The development of e-nose diagnostic detection methods using commercially-available e-nose instruments has a sound foundation with numerous examples of proven and documented successful applications. However, many new interesting and useful e-nose applications currently are under development and forthcoming in extant research efforts. For example, many important recent studies have been investigating the sensorial analysis of human breath to potentially provide rapid and dependable diagnoses of many human diseases based on the detection of bioindicator compounds and specific mixtures of aberrant VOCs in human breath samples. Some examples of these new research developments and discoveries of new e-nose theoretical applications are provided in the following discussion.

### 6.1. Asthma and Respiratory-Disease Detection

The investigation of human breath samples with conventional analytical methods has shown a correlation between specific VOCs or VOC mixtures and the occurrence of certain illnesses. Asthma, one of the most important lung diseases in clinical practice, currently is diagnosed and monitored by symptoms and physiological measurements. These tests have been internationally standardized and are considered to be reliable, but rather complex, time-consuming and not widely applicable in many cases. During the past decade, new options for diagnosis and monitoring lung diseases like asthma have been proposed. These include the determination of nitric oxide in exhaled air which appears to have a realistic potential of widespread application due to its simplicity [434,435]. It is also well recognized that the exhaled breath is composed by thousands of VOCs of which nitric oxide is only one of these. Thus, measurement of nitric oxide alone is not always sufficient to determine the identity or severity of a disease. For these reasons, Dragonieri *et al.* [250] in 2007 postulated the possibility of testing the capability of an electronic nose to discriminate exhaled breath of patients with asthma from healthy controls and to distinguish between different levels of asthma severity. The study was based on 40 nonsmoking adults subjects, divided into four groups based on a standard sample size (10 patients with mild asthma, 10 with intermittent-mild asthma, 10 with moderate-severe persistent asthma, and 10 in the healthy control group). The exhaled breath samples were analyzed by the Cyranose 320 e-nose. The result showed that this electronic nose could successfully discriminate between patients with mild asthma and moderately-severe asthma compared with controls, but it could not adequately discriminate between mild and severe asthma samples, indicated by an M-distance of only 1.23 between these two groups.

A recent study by Montuschi *et al.* [436] tested whether differences between alveolar and oropharyngeal/airway air could affect e-nose results when analyzing VOCs in exhaled breath. The study tried to ascertain whether an electronic nose could discriminate between patients with asthma and healthy subjects and to determine the best sampling protocol (alveolar vs oropharyngeal/airway air). They also compared the discrimination performance of the same electronic nose based on fractional exhaled iron nitric oxide (FeNO) in patients with moderately severe asthma. An experimental prototype electronic nose, containing eight QMV sensors coated with molecular films of metalloporphyrins designed at the University of Tor Vergata, gave the best results when performed on alveolar air. Moreover, the combination of electronic nose analysis of alveolar air and FeNO had the highest diagnostic performance for asthma (95.8%). Electronic nose analysis of alveolar air effectively discriminated between patients with asthma and healthy subjects in 87.5% of cases, much higher than for FeNO (79.2%), spirometry (70.8%), and a combination of FeNO and spirometry (83.3%).

Fens *et al.* [285] tested the possibility of using the electronic nose to discriminate between two diseases, Chronic Obstructive Pulmonary Disease (COPD) and asthma, which often exhibit overlapping clinical characteristics. The test groups consisted of 30 patients with COPD, 20 patients with asthma, 20 nonsmoking control subjects, and 20 smoking control subjects who participated in the study. Breath prints from patients with asthma were statistically well separated from patients with COPD at high levels of confidence (accuracy 96%, P < 0.001). Asthma patients also were statistically different from nonsmoking controls (95%) and from smoking control subjects (92.5%). Exhaled breath profiles of patients with COPD partially overlapped with those of smoking control subjects (accuracy 66%; P = 0.006).

The World Health Organization (WHO) has declared tuberculosis, a deadly infectious lung disease caused by the bacterium *Mycobacterium tuberculosis* (TB) that may attack other parts of the body, a global emergency. An estimated one-third of the world’s population is infected by the microbe. The majority of new infections take place in developing countries. The usual diagnostic method for TB involves the detection of acid-fast bacteria in the sputum by direct microscopy or by the Ziehl-Neelsen staining procedure which is often difficult. Pavlou *et al.* [196] investigated the possibility of detecting *Mycobacterium* species *in vitro* and *in situ* in sputum samples of TB-positive and TB-negative subjects. In this earlier study, the electronic nose successfully discriminated the TB-positive samples from the negative controls and between the different *Mycobacterium* species. In 2006, Fend *et al.* [321] investigated the potential of an electronic nose to detect the presence of *Mycobacterium* species in pure cultures and sputum samples from 280 patients with suspected TB and 50 non-TB controls in order to find a new fast and inexpensive TB-diagnostic method. A 14 CP-based electronic nose provided encouraging results. All unknown samples were correctly classified and the three different *Mycobacterium* species tested were closely clustered, but still effectively discriminated by statistical analysis.

Pneumonia is an inflammatory condition of the lungs caused by infections of bacteria, virus, fungi or other parasites, and by chemical or physical injury to the lungs. To date, there is no gold standard in the diagnosis of pneumonia. Diagnosis is based on a combination of all available data resulting from chest radiography, bronchoscopy, chest-computed tomography, and microscopic examination of the sputum. Hockstein *et al.* [229,230] proposed a novel method to diagnose pneumonia in patients receiving mechanical ventilation by means of the Cyranose 320 electronic nose. In both studies, this e-nose correctly discriminated between pneumonia-positive and pneumonia-negative patients. Good results also were obtained by Hanson and Thaler [231] based on e-nose analysis of 38 randomly selected mechanically-ventilated samples from 400 patients.

### 6.2. Biochemical Assays and Physiological Lab Tests

The application of electronic noses in biochemical and physiological assays for determining patient conditions and physiological states has been tested in several studies. Chen *et al.* [126] utilized magnetron sputtering and thermal oxidation titanium oxide thin films to determine blood compatibility in antithrombogenic investigations. Improvements in antithrombogenic properties of blood contacting biomaterials permitted a hybrid design of layers for biomedical applications such as artificial heart valves and stents. Xu *et al.* [125] developed a way to highly integrate different analytical operations such as sample preparation, separation, and detection into a single miniaturized device with the advantages of low cost, satisfactory analytical efficiency and flexibility in design. This integration of electrochemistry in microdevices for biochemical assays focused on *in situ* analysis, point-of-care testing and portable devices with simplicity, rapidity, high sensitivity, reduced power consumption, and sample/reagent economy. Easy microfabrication and integration of electrochemical components with multi-analytical functions was achieved at low cost. Such integrated microsystems likely will play an increasingly important role for analysis of small-volume biochemical tests.

### 6.3. Microbial Identifications in Culture and Body Fluids

Pavlou *et al.* [244] discriminated between headspace volatiles from complex broth cultures of some highly pathogenic gastroesophageal bacteria including *Staphylococcus aureus*, *Klebsiella spp*., *Helicobacter pylori* (the most common ulcer-causing pathogen) and *Enterococcus faecalis*. These bacteria were successfully identified at a diluted concentration of 10^7^ CFU mL^−1^. Other *in vitro* studies have involved recognition of several strains of two anaerobic bacteria (14 strains of *Clostridium* spp. and 12 strains of *Bacteroides fragilis*) and two fecal pathogens (*E. coli* and *Salmonella typhimurium*) by the use of an electronic nose [296,437]. Moens *et al.* [438] were able to identify ten clinically important microorganisms (*Pseudomonas aeruginosa*, *E. coli*, *Klebsiella pneumoniae*, *Enterobacter aerogenes*, *Proteus vulgaris*, *S. aureus*, *Streptococcus pneumoniae*, *E. faecalis*, *Candida albicans*, and *Aspergillus fumigatus*) using an electronic nose.

The development of microbial discrimination and classification of pathogenic bacteria by e-nose analysis of biological fluids of diseased patients also has been studied. Chandiok *et al.* [121] diagnosed bacterial vaginosis by e-nose analysis of vaginal swabs of 68 women attending a genito-urinary clinic. The conventional sensing approach was based on pH change, presence of clue cells, visual analysis of vaginal discharge, and human perception of amine-odor after addition of KOH. Hay *et al.* [439] dramatically improved the accuracy of the same pathological diagnosis using a commercially available electronic nose in two separate trials. The e-nose increased the accuracy and specificity of the diagnosis by 76–81% compared with Amsel criteria, and 77–83% compared with Gram-stain tests.

Lykos *et al.* [254] tested an electronic nose prototype designed and assembled by undergraduate students of the Illinois Institute of Technology on artificially-inoculated human blood cultures. After 12 to 36 hours incubation, the electronic nose could successfully distinguish between different bacteria species (*E. coli*, *Pseudomonas aeruginosa*, *S. aureus* and *E. faecalis*) causing bacteremia and septicemia. This was an improvement over conventional subculturing procedures done on diagnostic plate media. Fend *et al.* [188] did blood analysis with an electronic nose to monitor and quantify dialysis dosage in patients undergoing regular renal dialysis following kidney failure. The electronic nose was capable of discriminating between pre-dialysis from post-dialysis blood. This tool could be used for on-line dialysis monitoring and other potential applications as pathogen sensors [440].

### 6.4. Diagnosis of Local and Systemic Microbial Infections

Aathithan *et al.* [258] developed the Osmetech Microbial Analyzer (OMA) e-nose, an automated headspace analyzer fitted with a novel CP sensor array, for screening clinical urine specimens for bacteriuria by sampling urine headspace and multiple-detector PCA. The OMA distinguished artificially-infected urine samples from sterile controls in 534 clinical urine specimens, of which 21.5% had significant bacteriuria (containing >105 CFU of bacteria/ml). The accuracy and specificity of the OMA compared with conventional culture were 83.5 and 87.6%, respectively and this system was considered a promising automated system for rapid routine screening of urine specimens.

### 6.5. Progress of Healing, Physiological Monitoring, and Cellular Signaling

Wounds are injuries to body tissues caused by diseases, processes or physically-damaging events such as burns, punctures, lacerations, ulcers or surgical procedures. Wounds can become infected when microorganisms from the environment enter the open wound and multiply, sometimes leading to severe symptoms. The standard techniques for microbiological detection are surface swabbing to assess superficial infections and biopsies for deeper wounds which are invasive. Byun *et al.* [441] recently proposed a novel method for monitoring open wounds by means of an experimental e-nose containing a sensor array composed of commercially-available MOS sensors fabricated by Figaro (TGS 2602, TGS 2610), Japan, and experimental SnO_2_-based thin film MOS sensors doped with Au, Cr, or WO developed by INFM-CNR, Italy and the Semiconductor Physics Institute, Lithuania. Initial data indicated that infected patients could be distinguished from uninfected patients, but the system is currently being validated on a larger number of patient samples. Tian *et al.* [442] used a gas sensor array with six MOS sensors and one electrochemical gas sensor to identify seven species of pathogens common in wound infections *in vitro*. All seven pathogens were correctly identified and discriminated with an accuracy of 100% with this e-nose system.

Halitosis or bad breath has been shown to be due to various volatile sulfur compounds (VSCs), such as hydrogen sulfide, methyl mercaptan, and dimethyl sulfide produced by bacterial metabolism. Oral pathologic odor is caused by disease, pathological conditions and oral tissue malfunction. The organoleptic test has been used by medical practitioners for the evaluation of halitosis, but this test is somewhat subjective. Thus, the development of a halitosis measurement system for objective and reliable human breath evaluation is required. Commercially-available portable sulfide monitors using a gas sensor have insufficient sensitivity and selectivity to measure many VSCs. In 2004, Ito *et al.* [443] proposed a method for discriminating VSCs (hydrogen sulfide, methyl mercaptan, and dimethyl sulfide) using a Quartz Crystal Microbalance (QCM) sensor array combined with a preconcentrator. The results of PCA indicated that three types of VSCs were separated using the sensor array output pattern.

Tanaka *et al.* [425] tested the capability of the FF-1 electronic nose (composed of a pre-concentrator and an array of 6 MOS sensors) to discriminate the odors present in human breath of 49 patients complaining of oral malodor and 29 control subjects without halitosis. Tanaka’s work was continued by Nonaka *et al.* [444] who tried to express the clinically oral malodor intensity as an absolute value. The same e-nose was used and the absolute value of the malodor intensity was expressed as the vectorial sum of the malodor intensity of each category of gas. Yamada *et al.* [445] also used the same odor-sensing e-nose to assess the presence of putrescent odor produced in association with necrosis of dental pulp resulting from the presence of root canal bacterial infection. The odor from each mouth root canal of 39 patients was expressed as absolute odor by use of the diagnostic software called ASmell2. Pennazza *et al.* [446] focused on the design of breath-sampling procedures to optimize halitosis measurements and evaluate the threshold limits of the electronic nose towards biomarker compounds that play a key role in oral malodor manifestations.

Saraoğlu *et al.* [447,448] addressed the problem of anesthesia levels during surgeries. The effects of applied anesthesia on a patient, known as the depth of anesthesia, can change according to the chemicals used and characteristics of the patient. During a surgical operation, the depth of anesthesia is determined by an anesthetist, who has to continuously monitor and measure patient sensitivity and dosage level applied. A calibration defect or malfunction in the vaporizer equipment can bring the patient to a life-threatening critical condition. In this study, a prototype QMB electronic nose using sensors coated with phthalocyanine was developed with high sensitivity to sevoflurane, the anesthetic agent used in the study. The results of the first experiment indicated a success rate of 95% in the prediction of the effective anesthetic dose level applied. A latter experiment revealed a means for effectively reducing the delay in QMB response from 5 to 15 minutes down to 100 seconds.

### 6.6. Organ Transplant Rejection

Phillips *et al.* [192] evaluated a breath test for oxidative stress (based on breath alkanes) in heart transplant recipients and a mathematical model for predicting the probability of grade 3 heart-transplant rejections. The test was 100% accurate for grade 3 heart transplant rejection when a *P* value was ∃0.98, and 100% specific when the *P* value was #0.058. The alkane biomarkers were specific for the indication of oxidative stress related to heart allograft rejections. The potential use of e-nose evaluations and determinations of other types of organ transplant success based on breath or urine volatiles will be further explored in the coming years.

### 6.7. Cancer Detection

More than 20 years ago, Gordon *et al.* [449] and O’Neil *et al.* [293] suggested that the exhaled air of lung cancer patients could contain specific VOCs that could be associated with this disease. The very early diagnosis and prompt treatment of lung cancer has been shown to dramatically shift the 5-year survival rate from 14% to 48% [450]. The same results were obtained by Phillips *et al.* [64] who conducted GC-MS analysis of the exhaled breath of 108 lung cancer positive and negative patients and found 22 VOCs to be useful biomarkers of lung cancer.

Di Natale *et al.* in 2003 [283] probably first suggested the possibility of using an electronic nose for the early diagnose of lung cancer. They postulated that the bouquet of VOCs containing lung cancer biomarkers in the exhaled breath could be recognized and discriminated by means of an electronic nose with 10 QMB sensors. The results with 42 diseased and 18 non-diseased volunteers showed that this e-nose could correctly classify 100% of lung cancer patients, and 94% of healthy controls. Chen *et al.* [295] proposed a new prototype of an electronic nose based on a virtual array of SAW sensors with the sensor output analyzed using an image-recognition method and an improved ANN. This e-nose was able to detect eleven VOCs that were validated as markers of lung cancer, and subsequently obtained good preliminary results with hospitalized patients.

Machado *et al.* [234] tested the Cyranose 320 electronic nose on 14 individuals with bronchogenic carcinoma and 45 healthy control subjects to identify and discriminate between diseased and non-diseased patients. They also applied supporting vector-machine analysis of smell prints of exhaled breath to create a cancer-prediction model using a training set of exhaled gases from diseased and healthy controls. The results demonstrated effective discrimination between the two sets of samples. In the validation study, the electronic nose had almost 72% accuracy and 92% specificity for detecting lung cancer. The positive and negative predictive values were 66.6% and 94%, respectively.

Dragonieri *et al.* [451] was able to discriminate between patients affected by non-small cell lung cancer, chronic obstructive pulmonary disease, and healthy controls by means of the Cyranose 320 electronic nose. The results showed that the smell prints of patients with lung cancer could be discriminated from subjects with Chronic Obstructive Pulmonary Disease (COPD) and healthy controls. The breath of patients with chronic obstructive pulmonary disease, often associated with the development of the lung neoplasm, are now known to contain VOCs that are distinct from those in patients with lung cancer.

D’Amico *et al.* [452] investigated the possibility of using an electronic nose as a means of distinguishing lung cancer from other lung diseases. This study involved 98 patients grouped into three sample classes: those with non-small cell lung cancer, other lung diseases and healthy controls. The QMB gas sensor array utilized was the latest prototype version of the artificial olfaction system developed at the University of Rome “Tor Vergata” based on eight QMB sensors coated with metalloporphyrins. The discrimination between the control group and lung cancer was 85% accurate with a specificity of 100% and negative predictive value of 90%, due to four false negatives, and a positive predictive value of 100% with no false positives. The separation between cancer-affected patients from individuals having other lung diseases was as followings: 86% of correctly classified patients were determined with an accuracy of 92.8% and 78.6% for lung cancer and the other lung diseases, respectively.

Previously, the investigation of VOCs identified from the exhaled breath of lung cancer patients has revealed important biomarkers for this disease. However, biomarkers have not yet been identified for many other types of cancer. This problem is primarily due to the difficulty in determining which specific VOCs, among a vast myriad of possible compounds detected using GC-MS analysis, that is most correlated with a specific cancer type. Electronic noses promise to be very useful noninvasive tools for cancer detection because they are sensitive and relatively inexpensive for diagnostic applications and are able to discriminate between aroma signatures consisting of hundreds of unknown compounds. One of the first studies to investigate the possibility of discriminating between different tumor cell lines *in vitro* was performed by Gendron *et al.* [175]. They reported on the *in vitro* discrimination of tumor cell lines by use of an electronic nose. Cells from both tumor (adenocarcinoma, squamous cell carcinoma, mesothelioma) and normal healthy cell lines were suspended in saline solution and analyzed by the Cyranose 320 e-nose that distinguished between cancer cell lines derived from skin lesions. This same e-nose was later demonstrated to have good sensitivity towards VOCs emitted from skin portions of patients affected by melanomas.

The abnormal metabolisms of tumor cells cause alterations in the chemical composition of VOCs released from affected cells, resulting in differences in the aroma patterns of headspace volatiles which can be distinguishable by an electronic nose. In 2008, D’Amico *et al.* [453] tried to identify melanoma patients by measuring the VOCs emitted from dark skin lesions using the University of Rome “Tor Vergata” QMB e-nose prototype on 40 individuals. The results showed that this electronic nose correctly discriminated between diseased and healthy controls with an accuracy of about 80%, similar to the performance of more labor-intensive and time-consuming diagnostic methods.

Brain cancer was investigated by Kateb *et al.* [193] who assessed the possibility of detecting and differentiating between the aroma signatures of liver and heart organ tissue (from chickens) and differences between tumor cell lines of human glioblastoma and human melanoma tumors. The Jet Propulsion Laboratory’s electronic nose, based on 16 sensors with uniquely coated polymer-carbon black composite films, was able to distinguish between the two types of organ tissues and between the two types of tumor cell lines. In a similar study, Horvath *et al.* [454] used an e-nose to discriminate between different VOC signals emitted by ovarian carcinoma and normal tissues. This method showed an accuracy of 100% based on 15 samples of each tissue type. Bernabei *et al.* [455] investigated the possibility of an early and non-invasive diagnosis of urinary tract cancers using an electronic nose. Measurements of urine headspace VOCs were performed by means of a QCM e-nose with eight quartz crystal microbalance sensors coated with different metalloporphyrins. A total of 113 patients affected by various urological pathologies were tested and e-nose data were processed via PCA and discriminant analysis using partial least squares. Discrimination and differentiation between healthy and ill individuals was achieved between patients with prostate and bladder cancer. The headspace of urine samples of patients with urinary tract cancer was different from that of healthy individuals. These preliminary results confirm a correlation between urine headspace and urological pathologies, enhancing the future possibility of performing early cancer diagnoses.

### 6.8. Prosthetic e-Nose Interface Applications

Biddiss and Chau [200] utilized a Conductive Polymer (CP) e-nose as a sensor and actuator for the control of hand prosthetic applications. This is a very good example demonstrating the many possibilities for interfacing e-nose components with prosthetics for biomechanical applications. Hong *et al.* [190] evaluated an implanted piezoelectric floating-mass transducer for sensor-neurological interfacing for the correction of hearing loss. Many other e-nose integrations with other prosthetic devises and healthcare applications are under development and will no doubt be forthcoming in the future as new e-nose combinations with other mechanical-aid devices are conceptualized by biomechanical engineers.

## 7. Conclusions

The biomedical field is growing at such a rapid rate that the development of new e-nose applications in this field also is accelerating due to increased emphasis on research and development (R&D) of these sensor systems by companies that build diagnostic equipment for the healthcare industry. The increasing demand for problem solutions to many and varied medical needs of modern societies has increased the need for cheap, rapid, and reliable diagnostic systems that work in real-time. The increasing emphasis of research priorities and funding for the development of new e-nose technologies in the medical industry has been related to the higher cost of detection instruments needed for disease diagnoses, the increasing demand for such instruments in medical hospitals and research facilities, the greater availability of funding for instrument purchases, and the higher visibility of biomedical needs and new diagnostic discoveries [8]. The shift in emphasis of R&D activities of commercial companies that develop electronic noses also has been profit-motivated. Electronic nose instruments developed for diagnostic medical applications often are considerably higher priced and more lucrative for commercial development than for other industries.

The design of sensory systems found in nature often has served as model systems for the creation of instrumental olfactory systems that have led to some astonishing and innovative applications. The mammalian olfactory system is one of the most incredible natural systems that have been applied to machine olfaction and to olfactory diagnosis [456]. Thus, the inherent competent design of natural living systems often provide very effective models for the design of electronic noses for numerous sensing applications with both physiological and mechanical interactive components [132]. The development of bio-inspired devices, designed by the mimicry of natural biosensors, will no doubt lead to exciting new interactive and monitoring biomechanical e-noses that serve multiple bodily functions for individuals with impaired physiological systems. New sensor types and sensor coatings allow specialization and specificity in chemical detections. Reducing the number of detectors in the sensor array reduces the cost of portable e-noses needed for clinical and extraclinical diagnoses. The selection of sensor arrays by the utilization of large libraries of sensor types with different sensor coatings (sensitive to specific classes of VOCs) will facilitate the building of custom-designed e-noses for specific applications.

Advances in information technology and satellite communication systems, combined with novel intelligent sensors like e-noses, should result in better management of epidemic diseases such as tuberculosis (TB), autoimmune deficiency syndrome (AIDS), and other major diseases responsible for current world pandemics [457]. Thus, applying e-nose technologies should lead to better global disease control and reduced risk of spreading major infectious diseases around the world. By combining e-nose technologies with other advanced smart and adaptive engineering systems, it should be possible to create new tandem or interfaced systems with synergistic capabilities for disease diagnoses and other biomedical applications [458]. For example, electronic nose-related technologies using pattern recognition algorithms have been used in combination with blood flow spectra for disease classification in limb arteries [459], and for detection of toxin exposure associated with smoking [460]. Many other potential opportunities for combining e-nose technologies with other chemical-detection and mechanical devices such as analytical chemistry instruments [8], electronic tongues [131,138,461], biosensors [129–131,462–466], hydrogels [467,468], porous silicon [140,469], microbead arrays [137], various nanotechnologies [182,197], neurochips [134], mechanical drug-delivery systems [136,199,470,471], and image-analysis devices [472,473] have been emerging in recent years. The possibilities for the future development of entirely new electronic-noses and e-nose tandem devices for the healthcare industry are enormous. This potential will continue to increase as new innovative e-nose technologies are developed and become adapted for healthcare and biomedical applications.

Continuing improvements in effective clinical healthcare practices as we proceed into the 21st century increasingly will require that we make patient-treatment decisions based on multi-source information inputs, such as patient history, thorough physical examinations, symptomology, and physiological and biochemical real-time (rapid) diagnostic tests using e-nose or similar electronic devices. A final real-time confirmation of patient status and diagnosis using fast and reliable electronic devices is a very useful and necessary step to avoid mistakes in applying proper and appropriate patient treatments. Such a comprehensive approach to healthcare treatments previously has not been widely implemented hitherto and most current hospital and family clinic healthcare policies still use and rely on a “one target—one result” approach [456]. These non-confirmational practices will eventually be replaced as higher public expectations of medical standards and practices required more stringent procedures to not only avoid treatment mistakes, but also maintain favorable public relations and good reputations of healthcare institutions, and minimize cases of malpractice which greatly contribute to higher healthcare costs. The addition of electronic noses to help confirm patient status and diagnosis will contribute greatly to the development of more stringent criteria and practices for confirmation of diagnostic determinations prior to application of appropriate treatments.

There are several current limitations that have hindered the development of e-nose medical applications in the medical industry. One major problem is that there has not been sufficient trial in-hospital testing of e-nose instruments to determine the capabilities, feasibility and performance of these instruments for specific tasks (applications) within the operating practices of hospitals and other medical-care facilities. The slow willingness of some doctors and medical-services personnel to test and adopt new technologies, due to resistance to change from traditional methods, the existence of prejudices and biases against the capabilities of e-nose instruments, have precluded adequate efficacy testing and feasibility studies. Some hindrances to the advancement of e-nose applications have to do with the difficulty, often experienced by instrument developers, in identifying specific biomedical applications that would be the most feasible and useful in medical practice. This requires effective and specific input from medical practitioners on a regular basis. Many new medical applications have been developed and are in current use with very good e-nose efficacy data already available. Nevertheless, much more research is needed to develop and take full advantage of e-nose instruments to bring them to the full potential of capabilities for healthcare applications. With a vast amount of literature now available to demonstrate the theoretical and practical feasibility of using electronic noses in many diverse medical applications, the trick now will be to go the extra distance to begin to fine-tune these technologies for many practical and specific applications. This will require cooperative efforts and informative exchanges between researchers and medical practitioners. Once these difficulties and logistics are resolved, electronic-nose devices should be capable of solving many medical diagnostic problems and serving many of the future needs of the medical industry that have yet to be discovered.

## Figures and Tables

**Table 1.** Some early uses of descriptive aromas in diagnosing specific diseases and metabolic disorders associated with odors released from affected human tissues.

**Disease/Disorder**	**Body source**	**Descriptive aroma**	**References**
Acromegaly	Body	Strong, offensive	[22]
Anaerobic infection	Skin, sweat	Rotten apples	[23]
Azotemia (prerenal)	Urine	Concentrated urine odor	[24]
Bacterial proteolysis	Skin	Over-ripe Camembert	[23]
Bacterial vaginosis	Vaginal discharge	Amine-like	[23]
Bladder infection	Urine	Ammonia	[23]
Bromhidrosis	Skin, nose	Unpleasant	[25]
Darier’s disease	Buttocks	Rank, unpleasant odor	[26]
Diabetic ketoacidosis	Breath	Rotting apples, acetone	[26,27]
Congestive heart failure	Heart (portcaval shunts)	Dimethyl sulfide	[28]
Cystic fibrosis	Infant stool	Foul	[23]
Diabetes mellitus	Breath	Acetone-like	[23]
Diphtheria	Sweat	Sweet	[23,26,29]
Empyema (anaerobic)	Breath	Foul, putrid	[29]
Esophageal diverticulum	Breath	Feculent, foul	[26,29,30]
Fetor hepaticus	Breath	Newly-mown clover, sweet	[29]
Gout	Skin	Gouty odor	[26]
Hydradenitis suppurativa	Apocrine sweat glands	Bad body odor	[26]
Hyperhydrosis	Body	Unpleasant body odor	[26]
Hyperaminoaciduria (Oast-house Syndrome)	Infant skin	Dried malt or hops	[26,31]
Hypermethioninemia	Infant breath	Sweet, fruity, fishy, boiled cabbage, rancid butter	[23,26,29]
Intestinal obstruction	Breath	Feculent, foul	[26,29,30]
Intranasal foreign body	Breath	Foul, feculent	[23,29,30]
Isovaleric acidemia	Skin, sweat, breath	Sweaty feet, cheesy	[23,29,32,33]
Ketoacidosis (starvation)	Breath	Sweet, fruity, acetone-like	[29]
Liver failure	Breath	Musty fish, raw liver, feculent, mercaptans, dimethyl sulfide	[28,29]
Lung abscess	Sputum, breath	Foul, putrid, full	[23,26,29,30]
Maple syrup urine disease	Sweat, urine, ear wax	Maple syrup, burnt sugar	[23,26]
Phenylketonuria	Infant skin	Musty, horsey, mousy, sweet urine	[17,23,26]
Pneumonia (necrotizing)	Breath	Putrid	[23]
*Pseudomonas* infection	Skin, sweat	Grape	[23]
Renal failure (chronic)	Breath	Stale urine	[27]
*Rotavirus* gastroenteritis	Stool	Full	[23]
Rubella	Sweat	Freshly plucked feathers	[23]
Schizophrenia	Sweat	Mildly acetic	[34,35]
Scrofula	Body	Stale beer	[26]
Scurvy	Sweat	Putrid	[26]
Shigellosis	Stool	Rancid	[23]
Smallpox	Skin	Pox stench	[26]
Squamous-cell carcinoma	Skin	Offensive odor	[36]
Sweaty feet syndrome	Urine, sweat, breath	Foul acetic	[37]
Trench mouth	Breath	Halitosis	[29]
Trimethylaminuria	Skin, urine	Fishy	[23]
Tuberculosis lymphadenitis	Skin	Stale beer	[23]
Tubular necrosis (acute)	Urine	Stale water	[24]
Typhoid	Skin	Freshly-baked brown bread	[23,26,29]
Uremia	Breath	Fishy, ammonia, urine-like	[29]
Vagabond’s disease	Skin	Unpleasant	[26]
Varicose ulcers (malignant)	Leg	Foul, unpleasant	[38–40]
Yellow fever	Skin	Butcher’s shop	[26,29]

**Table 2.** Some major categories of potential electronic-nose applications within various sectors of the healthcare and biomedical industries.

**Healthcare sector**	**Specific application areas**	**References**
Biochemical tests	Biochemical assays	[125]
	Blood compatibility	[126]
	Cellular-signaling detection	[127]
	Clinical chemistry	[128]
Biomedical research	Biosensor development	[129–132]
	Sensory response	[133–138]
Chemical exposures	Contact or inhalation of toxic chemicals	[139,140]
	Biologically-active VOCs, harmful vapors	[141–148]
	Heavy metal exposure	[149]
Clinical biomechanics	Culture and *in vitro* medical analyses	[116]
Detection	Presence of toxic substances	[150–152]
Environmental hazards	Health hazards in the environment	[153–155]
Equipment mechanics	Operational life of healthcare equipment	[156,157]
Food contaminations	Microbial pathogens or toxins	[158–164]
	Chemical contaminants	[165]
Genetic testing	Presence of genetic anomalies/disorders	[166]
Immunology	Antibody monitoring	[167,168]
Infections	Local and systemic infections	[123,169]
Ingestion of toxins & poisons	Alcohol consumption and abuse	[170]
	Drug use and abuse	[171]
	Potable water contamination Pesticides	[172,173] [174]
Microbial culture	Discrimination of types or cell lines	[175]
	Identification of microbes	[176,177]
	Growth phases	[178–180]
Monitoring	Blood plasma glucose levels	[181–184]
	Hormone levels	[185,186]
	Human tissue activities	[187]
	Progress of dialysis	[188]
Organ dysfunctions	Enterogastric reflux	[189]
	Hearing loss	[190]
	Organ failures or abnormalities	[191]
	Organ transplant success	[192]
Pathology	Cancer detection	[193]
	Clinical disease diagnoses	[194,195]
	Pathogenic microbe detection in tissues	[196,197]
	Specific pathogen identifications	[198]
Pharmacology	Drug purity/contaminations, drug toxicoses	[8]
	Drug-delivery controls, drug effectiveness	[199]
Physical therapy	Prosthetic applications	[200]
Physiological conditions	Electrophysiological measurements	[201]
	Hydration, ketosis, oxidative stress	[202,203]
	Physiological disorders	[204]
Respiratory conditions	Oxygen-sensing measurements	[205]
Remote health care	Telemedicine	[206]
Waste disposal	Medical waste analysis	[207]
Wound & graft healing	Bacterial colonization, secondary infection	[208]
	Progress of healing	[209]
	Skin graft success	[210]

**Table 3.** Potential electronic-nose applications for human and animal disease diagnoses via detection of volatile biomarker indicator compounds.

**Disease/Disorder/Infection**	**Aroma sample source**	**Associated volatile biomarkers†**	**References**
Aerobic gram negative bacteria	Intraperitoneal fluid	Terpenes, ketones	[10]
Alcoholic fatty liver disease (AFDL)	Human breath	Acetaldehyde, isoprene, 3 other unidentified VOCs	[240]
Alcohol-induced hepatic injury	Human breath	Ethane, pentane (volatile alkanes)	[241]
Allograft rejection	Human breath	Carbonyl sulfide	[242]
Anaerobic bacterial infections	Intraperitoneal fluid	Acetic acid, butyric acid	[243]
	Urine	Isobutylamine, acetic acid, butyric acid	[106,244,245]
Asthma	Human breath	Pentane, ethane, 8-isoprostane, nitric oxide, leukotriene B4, prostaglandin E2	[246–253]
Bacteremia	Blood cultures	Specific complex mixture of VOCs	[254]
	White blood cell cultures	Acetaldehyde, hexanaldehyde	[255]
Bacterial infections (local)	Human pus	Isobutyric, isovaleric, & isocaproic acids	[256]
Bacterial vaginosis	Vaginal swabs	Specific complex mixture of VOCs	[257]
Bacteriuria	Urine	Specific complex mixture of VOCs	[258]
Breast cancer	Human breath	C4-C20 alkanes, monomethylated alkanes	[67]
Cardiopulmonary disease	Alveolar air	Ethanol, acetone	[259]
Cholera	Feces	p-menth-1-en-8-ol, dimethyl disulphide	[260]
Chronic hepatitis	Alveolar air	Methyl-mercaptan, dimethyl sulfide	[261]
Chronic obstructive pulmonary disease (COPD)	Human breath	NO, H_2_O_2_, aldehydes, 8-isoprostane, nitrotyrosine, Leukotriene B4, cytokines	[99,100,262–265]
Cystic fibrosis	Human breath	Leukotriene B4, interleukin-6, carbonyl sulfide, alkanes	[192,266]
Cystinuria	Human breath	Cadaverine, piperidine, putrescine, pyrrolidine	[42,267]
Diabetes mellitus	Human breath	Acetone, ethanol, methyl nitrate, complex VOCs	[226,268]
Eye infections	Cultures of ocular fluids	Specific complex mixture of VOCs	[176]
Foetor hepaticus	Human breath	Specific complex mixture of VOCs	[269]
Haematuria	Urine	Specific complex mixture of VOCs	[270]
Halitosis	Human breath	Hydrogen sulfide, methyl mercaptan, dimethyl sulfide	[271]
Hepatic cirrhosis	Alveolar breath	Dimethyl sulfide, hydrogen sulfide, mercaptans, volatile fatty acids	[272]
	Alveolar air	Dimethyl sulfide, acetone, 2-pentanone and 2-butanone	[273]
	chronic hepatitis	Methyl-mercaptan, dimethyl sulfide	[261,239]
Hepatic coma	Alveolar air	Methyl-mercaptan, dimethyl sulfide	[261,239,274]
Hepatic encephalopathy	Blood plasma, spinal fluid	3-methylbutanal	[72]
Histidinemia	Human breath	2-imidazolepyruvic acid, 2-imidazolelactic acid, 2-imidazoleacetic acid	[42]
Hyperglycemia	Human breath	Methyl nitrate, xylene, ethylbenzene	[183,268]
Inflammatory bowel disease (IBD)	Human breath	Pentane, ethane, propane	[73,275–277]
Isochemic heart disease, angina	Human breath	Alkanes, methylated alkanes	[278,279]
Ketosis	Alveolar air	Acetone	[76]
Leg ulcers & wound healing	Contact dressings	Specific complex mixture of VOCs	[280,281]
Liver cancer	Blood	Hexanal, 1-octen-3-ol, octane	[282]
Lung cancer	Human breath	Alkanes, monomethylated alkanes	[64,278]
	Human breath	Alkanes, aromatic compounds	[283]
	Human breath	Specific complex mixture of VOCs	[234,284–286]
Maple syrup urine disease	Human breath	2-oxoisocaproic acid	[42]
Metabolic disorders	Urine	Isovaleric acid	[287]
Necrotizing enterocolitis (NEC)	Feces	2-Ethyl-1-hexanol, fewer esters present in VOCs	[288]
Oxidative stress	Human breath	8-isoprostane	[247]
Phenylketonuria	Human breath	Phenylpyruvic acid, phenyllactic acid, phenylacetic acid	[42]
Renal dysfunction and failure	Urine	Specific complex mixture of VOCs	[270]
Respiratory infections	Human breath	Specific complex mixture of VOCs	[289]
Rheumatoid arthritis	Alveolar air	Pentane	[290]
Tyrosinemia	Human breath	p-hydroxyphenylpyruvic acid	[42]
Schizophrenia	Alveolar air	Pentane, carbon disulfide, ethane	[34,35,291]
Sweaty feet syndrome	Urine, sweat, human breath	Butyric acid, hexanoic acid; trans-3-methyl-2 hexenoic acid	[37]
Urinary tract infection	Urine	Isovaleric acid, alkanes	[245]
	Urine	Specific complex mixture of VOCs	[245,292]

† Bio-indicator compounds are volatile organic compounds (VOCs) that usually were initially identified and confirmed using gas chromatography-mass spectroscopy (GC-MS) or similar analytical equipment prior to development of corresponding e-nose reference libraries for detection.

**Table 4.** Potential electronic-nose applications for human and animal disease diagnoses via detection of volatile biomarker indicator compounds produced by bacterial pathogens that are causal agents of specific diseases.

**Pathogenic agent**	**Disease**	**Aroma sample source**	**Potential biomarkers**	**References**
*Campylobacter jejuni*	Gastroenteritis	Feces	Hexanal, (E)-2-octenal, pyrrole, ethyl ethanoate, methyl alcohol, 2-heptanone	[74]
*Clostridium septicum*	Intestinal disease	Culture from intestine	Isobutylamine, isopentylamine, ethylamine	[11]
*Enterococcus faecalis*	Gastroesophageal disease	Complex broth cultures	Terpenes, trimethylamine, ketones	[244]
*Escherichia coli*	Gastrointestinal diseases	Complex broth cultures	Terpenes, trimethylamine, ketones; ethanol	[244,320]
*Helicobacter pylori*	Stomach ulcers	Gastroesophageal cultures	Terpenes, trimethylamine, ketones	[244]
*Klebsiella pneumoniae*	Gastrointestinal diseases	Urine	Trimethylamine, ethyl acetate	[9]
*Mycobacterium tuberculosis*	Tuberculosis	Human sputum cultures	Specific complex mixture of VOCs	[17,27,302,321]
*Proteus mirabilis*	Urinary tract infections	Urine	Benzaldehyde, isobutyraldehyde, isovalaraldehyde; sulfides	[301,316]
	Urinary tract infections	Urine	Dimethyl sulphide, methyl mercaptan	[301]
	Urinary tract infections	Urine	Trimethylamine, ethyl acetate	[9]
	Urinary tract infections	Urine	Isobutanol, isopentyl acetate ketones, I-undecene	[287,317]
*Pseudomonas aeuroginosa*	Localized infections	Skin, lung	Butanol, methyl ketones (2- 2-nonanone, 2-undecanone), heptanone, 2-aminoaceto-phenone	[311,314,317]
*Pseudomonas perolens, P. taetrolens*	Food poisoning (eggs *etc.*)	Stomach	2-methoxy-3-isopropylpyrazine (MIPP)	[312,313]
*Staphalococcus aureus*	Localized & systemic infections	All parts of the body	Isobutanol, isopentyl acetate ketones, I-undecene	[318]
*Vibrio cholerae*	Cholera	Feces	p-menth-1-en-8-ol, dimethyl disulphide	[74]

† Bio-indicator compounds are volatile organic compounds (VOCs) that usually were initially identified and confirmed using gas chromatography-mass spectroscopy (GC-MS) or similar analytical equipment prior to development of corresponding e-nose reference libraries for detection.

**Table 5.** Nosocomial opportunistic pathogens detectable with e-nose instruments as disease-causing agents of both plants and humans in the hospital environment.

**Pathogenic microbe**	**Plant diseases**	**Human diseases**	**References**
**Bacterial pathogens**			
*Burkholderia cenocepacia*	Banana finger-tip rot	Increases mortality in cystic fibrosis patients	[324–326]
*Burkholderia cepacia*	Onion soft rot (sour skin)	Pulmonary infections particularly in cystic fibrosis patients, pneumonia, septicemia	[325,327,328]
*Burkholderia dolosa*	Opportunist plant pathogen	Increases mortality in cystic fibrosis patients	[325,326]
*Pantoea agglomerans*	Epidermal surfaces of thorny plants	Soft tissue and bone/joint infections	[329–332]
*Ralstonia syzygii*	Clove tree, banana	Blood-borne diseases	[333,334]
*Ralstonia taiwanensis*	Root nodules of *Mimosa* spp.	Opportunistic infections of cystic fibrosis patients	[335]
*Pseudomonas aeruginosa*	Many plants spp.	Pneumonia, urinary tract infections, respiratory infections, bone & joint infections, bacteremia	[336,337]
*Stenotrophomonas maltophilia*	Plant endophyte	pneumonia, urinary tract and blood stream infections	[338,339]
**Fungal pathogens**			
*Acremonium falciforme*	Saprophyte on plant debris	Cutaneous and subcutaneous mycetomas	[340,341]
*Aspergillus flavus*	Rots maize, peanuts	chronic sinusitis, keratitis, cutaneous aspergillosis, wound infections, osteomyelitis, aflatoxin toxicosis	[342]
*Aspergillus nidulans*	Bean, pea, and cucumber leaf spots, orange rot	Cutaneous and subcutaneous mycetomas	[343,344]
*Cercospora apii*	Celery, beet, tomato, lettuce, tobacco, potato leaf spots	Dermatophytic skin lesions and deformities	[345,346]
*Cladophyalophora carrionii*	Plant leaf spots	Chromomycosis skin infections	[347–350]
*Coccidioides immitis*		Respiratory tract, skin, bones, central nervous system infections	[351]
*Fonsecaea pedrosoi*		Chromomycosis skin infections	[347,352]
*Fusarium graminearum*	Wheat, barley, rice, head blight	Alimentary toxic aleukia, weight loss, convulsions, anorexia, impaired immunity	[353–355]
*Fusarium moniliforme*	Vegetable seed rot	Fusarium sepsis	[356,357]
*Fusarium oxysporum*	Vascular wilt of many plant spp.	Nondermatophytic onychomycosis, fusariotoxicosis, keratitis	[358,359]
*Fusarium solani, roseum*	Vascular wilt of solanaceous plants	Skin lesions, encephalopathy, panophthalmitis, keratitis	[360,361]
*Geotrichum candidum*	Fruit & vegetable saprophyte	Oral, vaginal, skin, & systemic infections	[362–364]
*Geotrichum capitatum*	Surfaces of plant foods (ingested)	Septicemia, meningitis, discitis, encephalitis, endocarditis, vertebral osteomyelitis, respiratory tract & gastrointestinal infections	[365]
*Phialophora verrucosa*	Wood and woody plant surfaces	Chromomycosis, cutaneous and subcutaneous infections	[347,366,367]
*Pseudallescheria boydii*	Saprophyte of vegetable oils and tissues	Cutaneous and subcutaneous mycetomas, systemic infections	[368–371]

**Table 6.** Biomedical applications developed using commercial and experimental electronic noses.

**E-nose model**	**Manufacturer**	**Sensors†**	**Biomedical application**	**References**
Bloodhound BH-114	Sensors Ltd.	14 CP	Detect bacteria and heavy metals in potable water	[172,418]
			Early detection of TB	[321,196]
			Detect spoilage bacteria and fungi in food	[419–422]
			Anaerobic bacteria	[296]
			*Helicobacter pylori*, gastroesophageal bacteria	[244]
			Urinary tract infections	[245]
			Early disease diagnosis, distinguish dermatophytes	[423]
Cyranose C320	Cyrano Sciences	32 CP	Bacterial & SA infections	[123,176]
			Pneumonia score prediction	[231]
			Upper respiratory tract infections	[232]
			Lung cancer detection	[234]
			Ear, nose, throat infections	[233]
			Bacteria identifications in blood and urine samples	[424]
DE 101	Experimental model	3 MOS	Detect renal dysfunction	[191]
EOS 835	Sacmi Imola	6 MOS	Toxigenic *Fusarium* in corn	[160]
FF-1	Experimental model	6 MOS	Oral malodor assessment	[425]
Fox 2000	Alpha MOS	6 MOS	Culture growth phases	[124]
			Diagnose diseases	[103]
Fox 4000	Alpha MOS	18 MOS	Flavor analysis to develop new drug formulations	[136]
JPL ENose	NASA	16 CP	Brain cancer detection	[193]
KAMINA	Karlsruhe Res. Cntr.	38 MOS	Ammonia and chloroform hazard-detection	[426]
MonoNoses	C-it	1 MOS	Identify bacterial pathogens	[177]
Moses II	Lenmartz Electronics	8 MOS, 8 QMB, 4 AGS	Discriminate coliform bacteria	[218]
PEN 2	Airsense Analytical	10 MOS	Clinical diagnoses, Identify bacteria	[427]
			Aflatoxin detection in corn	[162,216]
ZNose	Electronic Sensor Technology	1 SAW	Identify bacteria & yeasts	[428]

† Sensor type abbreviations: Amperometric Gas Sensors (AGS); Conducting Polymers (CP); Metal-Oxide Semiconductors (MOS); Quartz Microbalance (QMB); Surface Acoustic Wave (SAW). The numerical values before each sensor type indicate the number of each sensor type present within the instrument sensor array.
